# Altered regulation of septal peptidoglycan synthesis modulates β-lactam susceptibility in *Pseudomonas aeruginosa*

**DOI:** 10.1371/journal.pgen.1012319

**Published:** 2026-09-28

**Authors:** Jake Colautti, Alexander C. Anderson, Wyatt P. K. Clark, Lindsey S. Marmont

**Affiliations:** 1 Department of Biochemistry and Biomedical Sciences, McMaster University, Hamilton, Ontario, Canada; 2 Michael DeGroote Institute for Infectious Disease Research, McMaster University, Hamilton, Ontario, Canada; Michigan State University, UNITED STATES OF AMERICA

## Abstract

Bacterial cell division requires precise regulation of septal peptidoglycan (PG) synthesis by the essential SEDS-bPBP synthase FtsWI. Activation of FtsWI is thought to occur through an allosteric cascade initiated by the late-arriving divisome protein FtsN and transmitted via the FtsQ-FtsL-FtsB (FtsQLB) complex, but the molecular details of this process remain incompletely defined and differ across species. Here, using the conditional essentiality of *ftsN* in *Pseudomonas aeruginosa*, we identify substitutions in the non-enzymatic pedestal domain of FtsI that bypass the requirement for FtsN. These mutations restore cell division in Δ*ftsN* cells, suppress dominant-negative phenotypes of activation-defective *ftsL* alleles, and reduce cell length in an otherwise wild-type background, demonstrating that they alter FtsWI regulation to overcome the requirement for stimulation by both FtsN and FtsQLB. Mapping these mutations onto the *P. aeruginosa* FtsQLBWI structure reveals that they cluster on distinct surfaces of the pedestal domain. Because some of these substitutions have been identified in clinical *P. aeruginosa* isolates with increased aztreonam resistance, we next asked whether these regulatory substitutions alter β-lactam susceptibility. Variants at the FtsI-FtsL interface modestly reduce susceptibility to multiple β-lactam antibiotics, whereas a variant on the opposite face of the pedestal domain produces striking FtsN-dependent hypersusceptibility to the same molecules. These findings show that the FtsI pedestal domain plays a central role in the regulation of FtsWI and reveal a previously unrecognized relationship between divisome regulation and β-lactam susceptibility. More broadly, our work identifies the pedestal domain as a regulatory hub that integrates multiple inputs controlling septal PG synthesis and highlights its potential relevance to β-lactam resistance in clinical *P. aeruginosa* isolates.

## Author summary

Bacterial cell division is driven by the coordinated activation of septal peptidoglycan synthesis, but the regulatory mechanisms that govern this process remain poorly understood. Here we identify mutations in the non-enzymatic pedestal domain of the septal transpeptidase FtsI that bypass the requirement for the activation factor FtsN in *Pseudomonas aeruginosa.* These substitutions reveal that the pedestal domain integrates regulatory inputs from both FtsN and the FtsQLB complex to control FtsWI activation. Notably, substitutions at the FtsI-FtsL interface modestly reduce susceptibility to multiple β-lactam antibiotics that specifically target FtsI, whereas a mutation on the opposite face renders *P. aeruginosa* hypersusceptible to these agents. These findings uncover a previously unrecognized link between divisome regulation and β-lactam susceptibility and suggest that altered activation of septal peptidoglycan biosynthesis represents a noncanonical contributor to antibiotic resistance in *P. aeruginosa*.

## Introduction

Most bacteria are encased in a peptidoglycan (PG) cell wall composed of glycan strands crosslinked by short peptide bridges [1]. This highly conserved exoskeleton defines cell shape and protects cells from osmotic lysis [2]. Because PG biosynthesis and recycling are essential for growth, many widely used antibiotics target these processes. A detailed understanding of how bacteria build and remodel the PG layer is therefore central to developing new agents that act on this structure.

PG is synthesized from the membrane-bound precursor lipid II by two core enzymatic activities. A PG glycosyltransferase polymerizes glycan strands into the growing cell wall, and a transpeptidase crosslinks these polymers through their peptide stems [3]. In most bacteria, these reactions are carried out by two distinct classes of PG synthases. Class A penicillin-binding proteins (aPBPs) contain both PG glycosyltransferase and transpeptidase activities within a single polypeptide [4], whereas the second type of PG synthase consists of a complex formed by a SEDS (shape, elongation, division, sporulation) protein paired with a cognate class B PBP (bPBP). In these PG synthase complexes, the SEDS protein carries out the PG glycosyltransferase reaction and the bPBP performs transpeptidation [5–8].

Incorporating new cell wall material requires transient weakening of PG integrity, which can be lethal if not precisely controlled [1,9]. This coordination is especially critical at the division septum, where a multiprotein complex known as the divisome couples PG synthesis with membrane invagination and the production of additional surface glycans required for cell division and separation [10]. Both the essential SEDS-bPBP pair FtsW-FtsI (FtsWI) and the non-essential aPBP PBP1b contribute to septal PG biogenesis. PBP1b is thought to reinforce the developing septum, whereas FtsWI functions as the primary septal PG synthase within the divisome [11].

In Gram-negative bacteria, septal PG synthesis is coordinated by a set of essential divisome proteins that assemble at midcell through a largely linear recruitment pathway [12–19]. The arrival of the protein FtsN is thought to initiate cytokinesis by triggering an allosteric signaling cascade that stimulates PG polymerization. Although the mechanism remains incompletely resolved, FtsN likely promotes divisome activation through its interactions with FtsA in the cytoplasm and with the membrane-embedded FtsQ-FtsL-FtsB (FtsQLB) subcomplex, which together activate the FtsWI synthase [20–23]. In *Escherichia coli*, FtsN uses an essential periplasmic α-helix to bind and activate FtsQLB, inducing a conformational change that enhances FtsW-mediated glycan polymerization [20,24,25].

A recent structure of the *P. aeruginosa* FtsQLBWI complex showed that the small α-helical proteins FtsL and FtsB contact the pedestal domain of FtsI, although FtsN does not stably associate with this assembly *in vitro [*20,26]. The pedestal domain is a conserved, non-enzymatic region of bPBPs, and accumulating evidence implicates it as an allosteric control point for SEDS-dependent PG polymerization [5]. Consistent with this idea, substitutions within the *E. coli* FtsI pedestal domain can bypass the requirement for both FtsN and FtsQLB-mediated activation [27,28]. These structural and genetic observations support a model in which signals initiated by FtsN are transmitted through FtsQLB to the FtsI pedestal domain to stimulate FtsWI activity [5,27,28]. However, the FtsN motif needed for activation in *E. coli* is not conserved in *P. aeruginosa*, and growth conditions have been reported under which *P. aeruginosa* can divide without FtsN [20]. This divergence suggests that the mechanism by which FtsN activates the divisome is not universal among Gram-negative bacteria.

Given these potential differences, we exploited the conditional essentiality of *ftsN* in *P. aeruginosa* to develop an unbiased genetic strategy to examine how FtsI regulates FtsW during cell division. We isolated substitutions in the FtsI pedestal domain that bypass the requirement for FtsN, restoring viability and normal morphology to cells lacking this divisome component. These substitutions also reduce cell length in an otherwise wild-type background, indicating that they alter cell division even in the presence of FtsN. They further suppress the cell division defects caused by dominant-negative FtsL alleles that fail to activate PG synthesis [20], indicating that our FtsI mutations overcome the need for activation by either FtsN or FtsL. Two of these substitutions have previously been identified in aztreonam-resistant clinical isolates from patients with cystic fibrosis, and we show that each mutation is sufficient to reduce susceptibility to multiple clinically important β-lactam antibiotics. This suggests that altered regulation of FtsWI can contribute to reduced β-lactam susceptibility. By contrast, a third substitution on the opposite face of the pedestal domain causes strong hypersusceptibility to aztreonam, and this phenotype is lost in an *ftsN* deletion background, consistent with FtsN-dependent activation of PG polymerization driving hypersusceptibility. Together, these results identify the FtsI pedestal domain as a regulatory hub that integrates multiple divisome activation signals, with distinct consequences for cell division and β-lactam susceptibility.

## Results

### FtsI pedestal domain substitutions bypass the requirement for FtsN

To better understand how FtsN stimulates septal PG synthesis in *P. aeruginosa*, we sought to identify substitutions in FtsI that bypass the requirement for upstream activation signals. Although FtsN is essential in many bacteria, including *E. coli* [29], it is dispensable for growth of *P. aeruginosa* on rich media supplemented with 5% (w/v) sucrose [20]. While it is not clear why sucrose supports the growth of this mutant, this effect may be due in part to osmotic protection, as FtsN remains essential for *P. aeruginosa* growth on media lacking sucrose [20]. We reasoned that this conditional essentiality could be used to isolate FtsI variants that support cell division without FtsN, and that some such variants might also circumvent the requirement for activation by FtsQLB. To test this idea, we introduced a mutagenized, IPTG-inducible *ftsI* library into a ∆*ftsN* strain and selected for transformants on Vogel-Bonner minimal medium (VBMM) supplemented with IPTG, thereby enriching for FtsI variants capable of restoring growth under these restrictive conditions.

Sequencing of 30 independent *ftsI* clones revealed alleles containing multiple amino acid substitutions (S1 Appendix**, Table A**). Because individual plasmids often carried several mutations, we reconstructed alleles encoding single substitutions to identify the changes responsible for bypassing FtsN. We prioritized substitutions isolated more than once, reasoning that recurrence likely reflected a direct role in restoring growth in the ∆*ftsN* background. Of the eight single substitutions tested, four (F167L, N212D, N212K, and P215L) supported growth of the ∆*ftsN* strain on VBMM in an IPTG-dependent manner, demonstrating that these alleles bypass the requirement for FtsN (S1 Appendix**, Fig. A**). Notably, all four substitutions map to the FtsI pedestal domain (Fig 1A), a non-enzymatic region implicated in allosteric control of SEDS-bPBP PG synthases [5,28].

**Fig 1:**
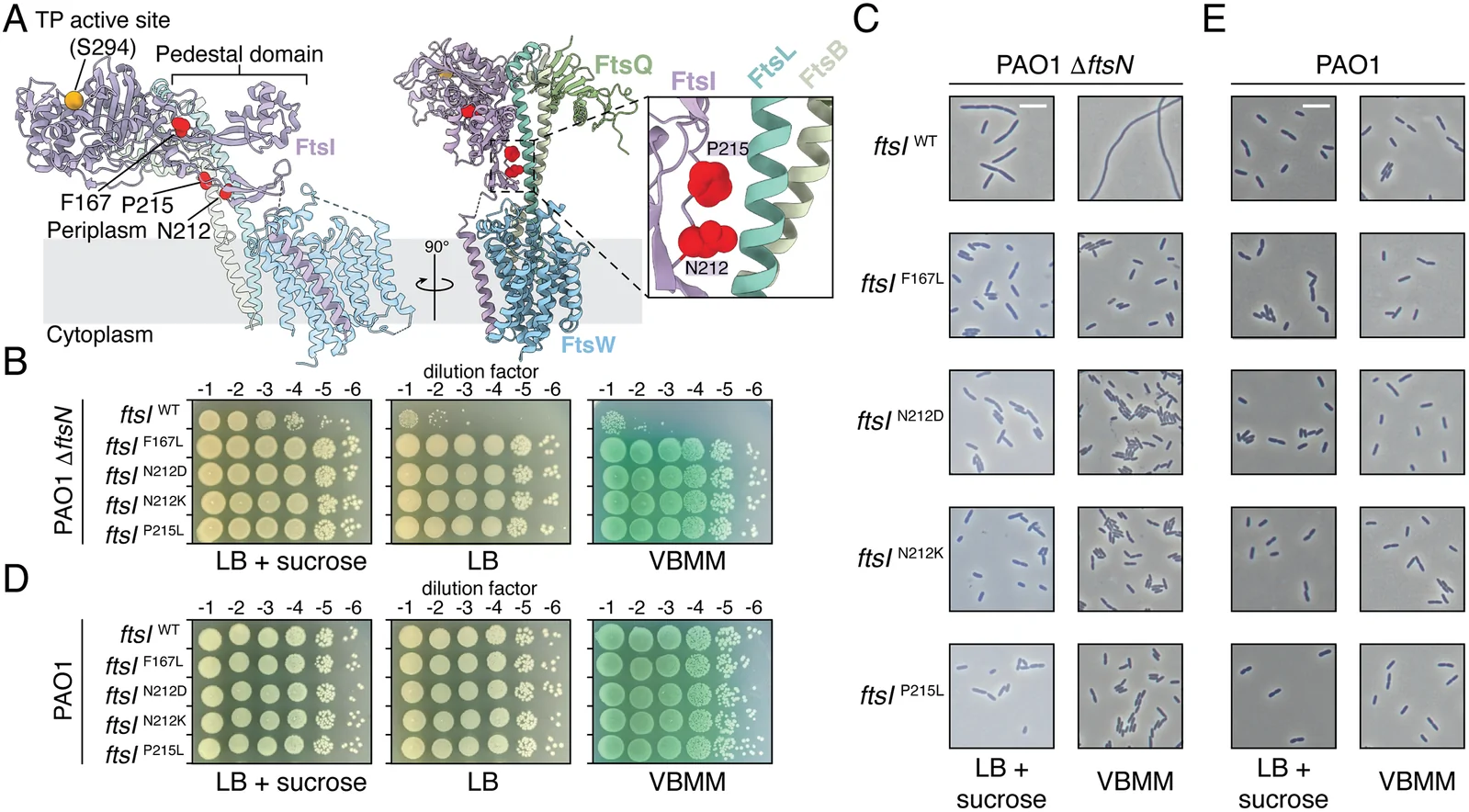
Substitutions in the pedestal domain of FtsI bypass the essential divisome protein FtsN. **A)** Cryo-EM structure (PDB 8BH1) of *P. aeruginosa* FtsQLBWI. Proteins are viewed from the plane of the membrane in cartoon representation. FtsI (purple), FtsW (blue), FtsQ (dark green), FtsL (turquoise) and FtsB (beige) are shown with the positions of FtsN-bypassing substitutions (red) and the catalytic residue of FtsI’s active site (yellow) depicted in sphere representation. The approximate position of the cytoplasmic membrane is depicted in gray. **B)** Tenfold serial dilutions of *P. aeruginosa* PAO1 ∆*ftsN* cells encoding the indicated *ftsI* alleles at the native locus plated on LB containing 5% (w/v) sucrose, LB, or VBMM. **C)** Phase-contrast micrographs of *P. aeruginosa* PAO1 ∆*ftsN* cells as in (B) harbouring the indicated *ftsI* alleles at the native chromosomal locus grown in LB containing 5% (w/v) sucrose or VBMM. **D)** Tenfold serial dilutions of *P. aeruginosa* PAO1 cells encoding the indicated *ftsI* or *ftsL* alleles at the native locus plated on LB containing 5% (w/v) sucrose, LB, or VBMM. **E)** Phase-contrast micrographs of *P. aeruginosa* PAO1 cells as in (D) harbouring the indicated *ftsI* or *ftsL* alleles at the native chromosomal locus grown in LB containing 5% (w/v) sucrose or VBMM. In panels B-E, bacteria were grown at 37°C. In panels C and E, scale bar represents 5 µm. Representative images of 2 independent experiments are shown in B-E.

We also assessed whether these *ftsI* alleles bypass FtsN when encoded at the chromosomal *ftsI* locus rather than overexpressed from a plasmid. As reported previously, *P. aeruginosa* ∆*ftsN* strains exhibit severe growth defects on LB or VBMM, but this impairment was eliminated in strains encoding any of the four single amino acid FtsI variants (Fig 1B). Consistent with the role of FtsN in triggering septal PG synthesis, ∆*ftsN* cells form long filaments when grown in VBMM (Fig 1C). Each bypass allele suppressed this filamentation phenotype, indicating that these FtsI variants support cell division in the absence of FtsN and independently of normal divisome activation (Fig 1C). Together, these findings demonstrate that substitutions in the FtsI pedestal domain can overcome the requirement for FtsN-mediated stimulation of septal PG synthesis.

Previous studies of the elongasome bPBP PBP2 showed that its pedestal domain undergoes a hinge-like conformational change that regulates its enzymatic activity [5,30]. Consistent with a similar mechanism in *P. aeruginosa* FtsI, expression of two independently engineered FtsI variants, each containing a distinct pair of cysteine substitutions designed to form an artificial disulfide bond and lock the pedestal domain in the closed conformation, inhibited growth, and this defect was relieved under reducing conditions (Fig 2A, 2B). Because the dominant-negative phenotypes of these variants indicate that the mutant proteins retain sufficient function to interfere with the native divisome complex, the growth defects are unlikely to result from a simple loss of protein function. These findings support a model in which FtsWI activity is controlled by allosteric movements within the FtsI pedestal domain, and together with prior work implicating this region in bPBP regulation, our results suggest that the substitutions recovered in our screen bypass FtsN by activating PG synthesis [5].

**Fig 2:**
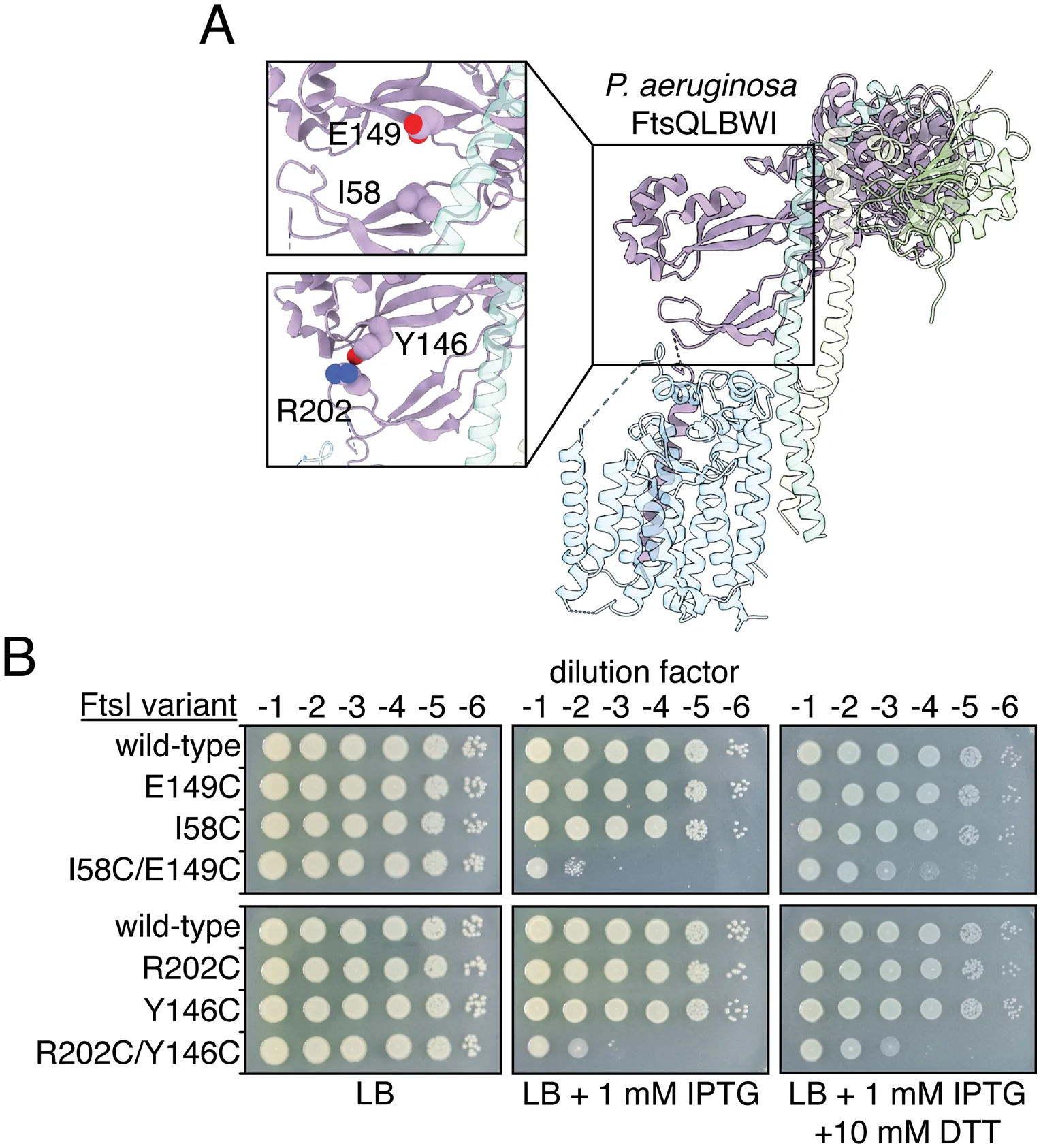
Artificial disulfide bonds engineered into the FtsI pedestal domain inhibit growth of *P. aeruginosa.* **A)** Cryo-EM structure (PDB 8BH1) of the *P. aeruginosa* FtsQLBWI complex, with the positions of residues mutated to cysteine to introduce artificial disulfide bonds depicted as spheres in the inset. Colouring of the complex is as described in Fig 1(A). B) 10-fold serial dilutions of *P. aeruginosa* PAO1 strains harbouring plasmids encoding wild-type *ftsI* or *ftsI* alleles bearing the indicated amino acid substitutions plated on LB, LB containing 1 mM IPTG to induce *ftsI* expression, or LB containing 1 mM IPTG and 10 mM dithiothreitol (DTT). Representative images of two independent experiments are shown.

### FtsI pedestal domain substitutions reduce cell length

In addition to the FtsI substitutions identified in our screen, previous work has shown that mutations in FtsL and FtsB can also bypass FtsN, and characterization of these so-called superfission alleles provided early genetic evidence that FtsQLB activates septal PG synthesis [20–22]. Genetic and microscopy analyses have shown that these mutants display temperature-sensitive lethal phenotypes distinct from cell filamentation. These phenotypes are thought to result from premature initiation of cell constriction, which reduces cell length without producing the filamentation characteristic of defective septation [21,22]. Although most studies were conducted in *E. coli*, a previous study demonstrated that the superfission substitution in *P. aeruginosa* FtsL (Q65K) similarly suppresses the growth and morphology defects of a ∆*ftsN* strain and displays a reduced cell length compared to the wild-type strain at 42°C [20].

Given that our FtsI substitutions also bypass FtsN, we asked whether these variants alter cell length in an otherwise wild-type background, as observed for previously characterized superfission alleles. To test this idea, we introduced each allele into the chromosomal *ftsI* locus of wild-type *P. aeruginosa* and assessed growth and morphology. All four alleles supported growth in an otherwise wild-type background on LB with or without sucrose and on VBMM (Fig 1D, 1E), indicating that the substitutions do not compromise FtsI function under these conditions. However, cell length was modestly but significantly reduced for each of these mutants in LB with sucrose and VBMM (S1 Appendix**, Fig. B**). We next compared these strains to the known FtsL superfission allele FtsL(Q65K) at 42°C. While growth was unaffected at this temperature (Fig 3A), substitutions in FtsI caused a marked reduction in cell length comparable to the FtsL superfission allele (Fig 3B-3C). Together, these findings demonstrate that the pedestal domain substitutions identified in our screen (hereafter FtsI*) reduce cell length in an otherwise wild-type background, consistent with altered regulation of cell division.

**Fig 3:**
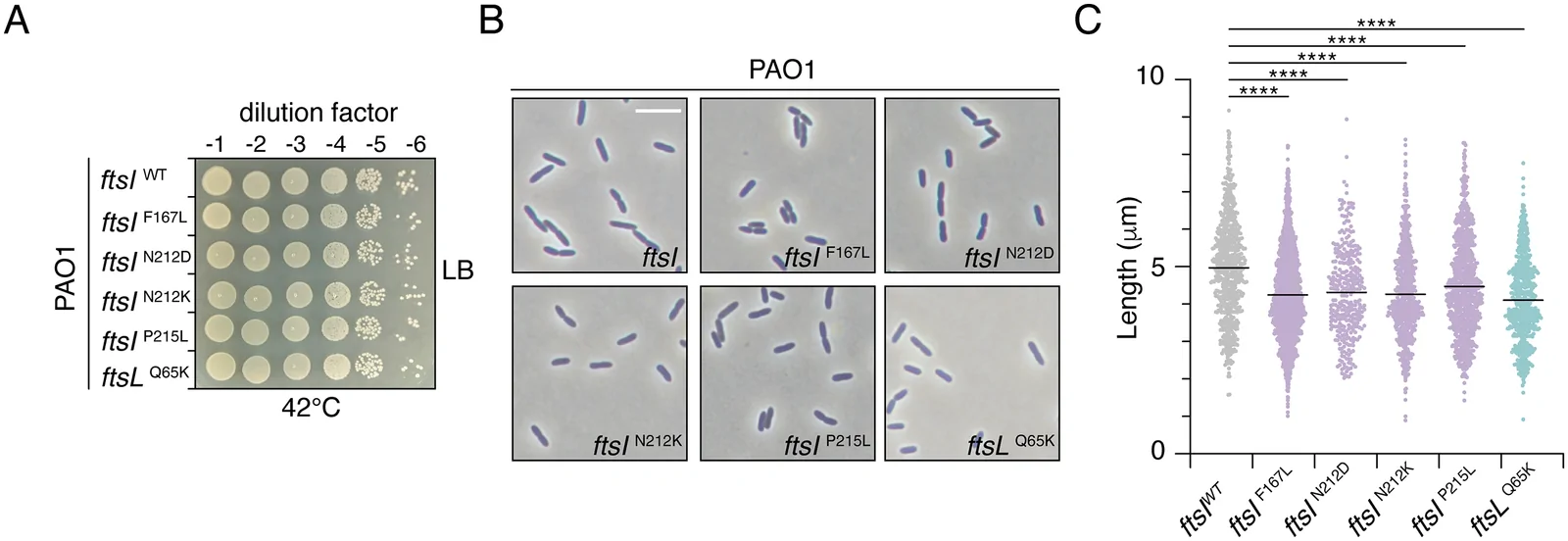
FtsI pedestal domain substitutions cause a reduction in cell length. **A)** Tenfold serial dilutions of *P. aeruginosa* PAO1 strains encoding the indicated *ftsI* or *ftsL* alleles at the native chromosomal locus plated on LB and grown at 42°C. **B)** Phase-contrast micrographs of *P. aeruginosa* PAO1 strains encoding the indicated *ftsI* or *ftsL* alleles at the native chromosomal locus grown in LB at 42°C. Scale bar represents 5 µm. In A and B, images are representative of two independent experiments. **C)** Quantification of cell length from micrographs from the experiment shown in **(B)**. Bars represent mean. Statistical significance was determined by one-way ANOVA, **** = *p* < 0.0001.

### FtsI* substitutions overcome the requirement for activation by FtsQLB

Having identified FtsI substitutions that bypass FtsN, we next examined their structural context within the recently solved *P. aeruginosa* FtsQLBWI cryo-EM structure [26]. N212 and P215 form part of the interaction interface between FtsI and FtsL (Fig 4A**, red spheres**), consistent with prior evidence that this region mediates FtsQLB-dependent activation of FtsWI [20,26]. This same interface contains residues altered in superfission FtsL variants such as Q65K (Fig 4A**, yellow spheres**), as well as previously established dominant-negative FtsL mutants (R38D, E64K, and S66D) [20] that localize to the division septum normally but fail to activate PG synthesis, resulting in cell filamentation and loss of viability (Fig 4A**, indigo spheres**).

**Fig 4:**
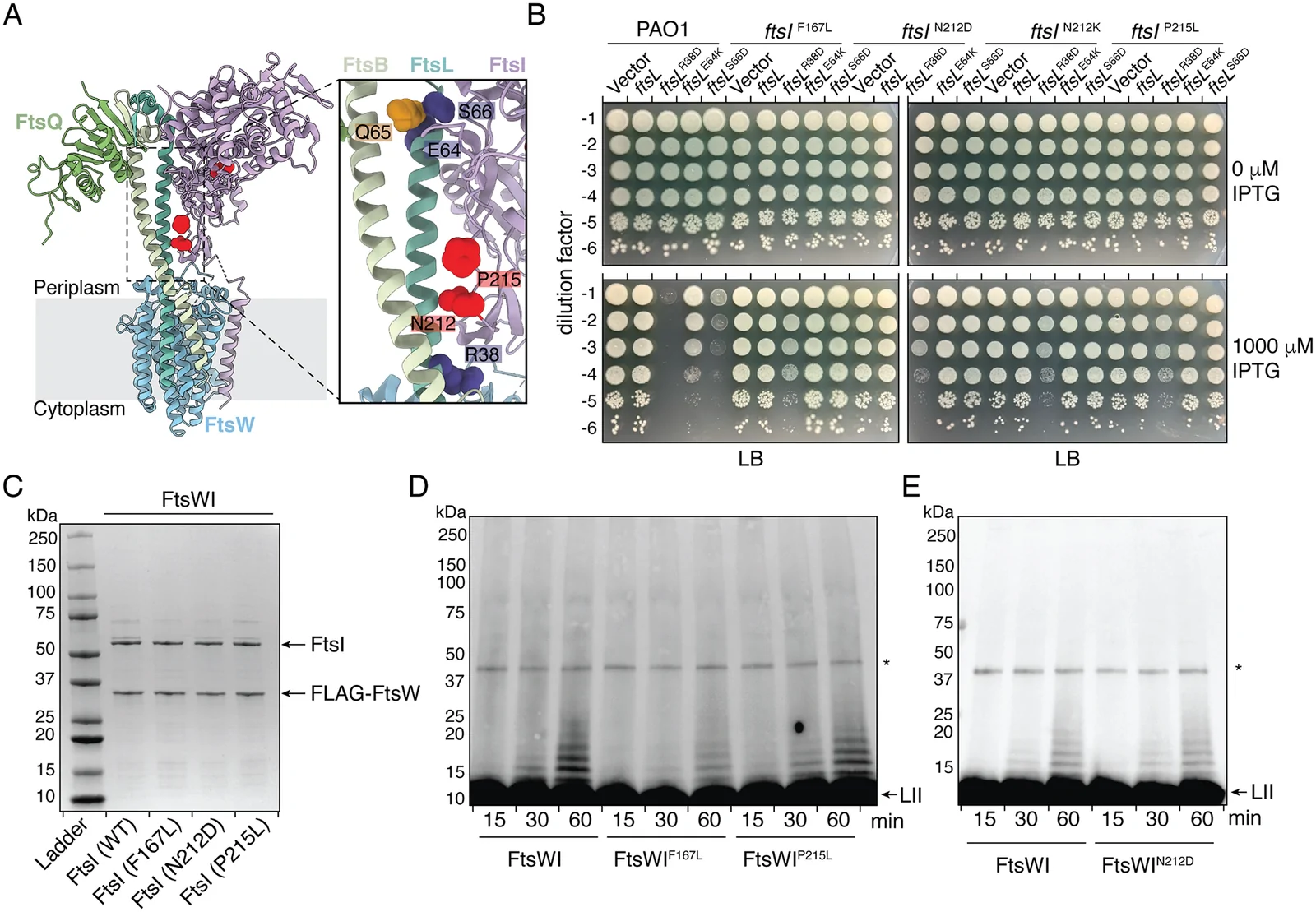
FtsI* variants overcome the requirement for FtsQLB activation *in vivo* but do not display enhanced PG glycosyltransferase activity *in vitro.* **A)** Cryo-EM structure (PDB 8BH1) of *P. aeruginosa* FtsQLBWI, shown in cartoon representation with the approximate position of the membrane depicted in gray. Colouring is as described in Fig 1(A). Inset shows the proximity of the FtsI* variants to the previously characterized FtsL residues involved in enhancing (Q65, yellow spheres) or inhibiting (R38, E64, S66, indigo spheres) cell division. FtsI* variants (red spheres) and FtsL residues (yellow and indigo spheres) are depicted in sphere representation. **B)** Tenfold serial dilutions of *P. aeruginosa* PAO1 cells encoding the indicated *ftsI* alleles at the native chromosomal locus and harbouring expression plasmids encoding the indicated FtsL variants. Dilutions were plated on LB agar containing 1000 µM IPTG to induce *ftsL* expression from the P_lacUV5_ promoter. **C)** Coomassie-stained SDS-PAGE gel of purified FLAG-tagged FtsW co-expressed with the indicated FtsI variants. D-E) PG glycosyltransferase assay time courses using the same normalized FtsWI complexes shown in **(C)**. Purified FtsWI complexes (0.5 µM) were incubated with *E. faecalis* lipid II (LII) (10 µM) and cephalexin (200 µM) to block cross-linking. The resulting glycan polymers were subsequently labelled with biotin-D-lysine using *S. aureus* PBP4 and detected by Western blotting using IRDye-labelled streptavidin. The asterisk indicates PBP4, which itself becomes labelled. Representative images from three independent experiments using at least two independent protein purifications are shown.

The proximity of the FtsI substitutions at N212 and P215 to these regulatory residues suggested that FtsI* might bypass the requirement for FtsQLB-mediated activation. If so, these variants should suppress the dominant-negative phenotypes of activation-defective FtsL alleles. To test this, we introduced plasmids expressing either wild-type *ftsL* or the dominant-negative *ftsL* alleles under IPTG control into *P. aeruginosa* strains encoding each FtsI* substitution at the native locus. As reported previously, expression of FtsL^R38D^, FtsL^E64K^, or FtsL^S66D^ inhibited growth and induced pronounced filamentation in wild-type cells (Figs 4B **and** S1 Appendix **Fig C panel A**). In contrast, strains carrying any of the FtsI* substitutions tolerated expression of these dysfunctional FtsL proteins and maintained normal rod-shaped morphology (Figs 4B **and** S1 Appendix **Fig C, panel A**). Quantification of these cells confirmed that expression of activation-defective FtsL variants increased cell length relative to wild-type FtsL (S1 Appendix**, Fig C, panel B**). In strains carrying FtsI* substitutions, this increase in cell length was suppressed (S1 Appendix**, Fig C, panel B**), consistent with the restoration of growth observed in these backgrounds. These observations indicate that the pedestal domain substitutions allow FtsWI to function without normal activation by FtsQLB.

### FtsI* substitutions do not increase FtsWI activity *in vitro*

One possible explanation for the ability of the pedestal domain substitutions to bypass FtsN and overcome the requirement for FtsQLB *in vivo* is that these variants intrinsically increase FtsWI activity, thereby increasing PG polymerization independently of upstream signals. To test this idea directly, we purified the mutant FtsWI complexes (Fig 4C) and measured their glycosyltransferase activity *in vitro* (Fig 4D, 4E**)** using a well-established PG glycosyltransferase assay [6,20]. As reported previously, wild-type FtsWI generates only short glycan polymers, consistent with its intrinsically weak polymerase activity in the absence of FtsQLB stimulation [20]. Each FtsWI* complex produced glycan strands of similar length and abundance to the wild-type enzyme complex (Figs 4D, 4E**, and** S1 Appendix**, Fig. D**). These results indicate that the pedestal domain substitutions do not intrinsically enhance FtsWI catalytic activity *in vitro*. Despite the ability of these FtsI* variants to bypass activation signals *in vivo*, their regulatory effects may depend on features of the intact divisome that are not recapitulated in our *in vitro* assay. Consistent with this possibility, the previously characterized FtsL(Q65K) superfission variant similarly does not increase the activity of the purified FtsQLBWI complex despite its activation phenotype *in vivo* [20].

### FtsI* substitutions reduce susceptibility to divisome-targeting β-lactam antibiotics

Because of its essential role in bacterial physiology, the cell wall is a major target of antibacterial therapeutics. β-lactam antibiotics, which irreversibly inhibit the transpeptidase activity of both aPBPs and bPBPs and thereby induce a futile cycle of PG polymerization and degradation, remain among the most widely used antimicrobial agents [31,32]. Aztreonam, a monobactam that selectively inhibits FtsI, disrupts septal PG homeostasis and is commonly used to treat chronic *P. aeruginosa* infections in individuals with cystic fibrosis [33–36]. In aztreonam-resistant clinical isolates, most *ftsI* mutations cluster within the TP domain near the drug-binding pocket and are thought to impair antibiotic binding [36–38]. Some resistant isolates, however, encode substitutions in the pedestal domain, far from the aztreonam-binding site, although whether these mutations contribute directly to resistance has not been tested [36]. Notably, two residues identified in our FtsN-bypass screen, N212 and P215, are mutated in such isolates, suggesting a possible link between β-lactam resistance and regulatory mechanisms controlling septal PG synthesis. These substitutions were identified among clonally related isolates from patients with cystic fibrosis treated with inhaled aztreonam in the AIR-CF5 clinical trial [36]. Among 64 isolate pairs selected for at least a fourfold difference in aztreonam MIC, one isolate contained an FtsI N212T substitution, and another contained P215L.

Because clinical isolates contain numerous chromosomal mutations that may influence antibiotic susceptibility, including changes in the levels of the AmpC β-lactamase and the outer membrane porin OprD, the specific contribution of pedestal domain substitutions to aztreonam resistance is unclear [36]. To test whether these substitutions directly affect susceptibility, we examined the growth of *P. aeruginosa* strains encoding each ftsI* allele at the native chromosomal locus in the presence of increasing aztreonam concentrations. Strains carrying *ftsI*^N212D^, *ftsI*^N212K^, or *ftsI*^P215L^ displayed modestly reduced aztreonam susceptibility relative to wild-type, demonstrating that these substitutions are individually sufficient to alter aztreonam susceptibility (Fig 5A). A similar pattern was observed when ftsI* strains were exposed to ceftazidime or piperacillin (Fig 6A, 6B), two clinically important β-lactams that preferentially target FtsI and are widely used in both directed anti-Pseudomonal therapy and empirical treatment regimens [39–42]. In contrast, none of the substitutions altered susceptibility to carbenicillin (Fig 6C), which primarily targets PBPs other than FtsI, indicating that the susceptibility phenotype reflects an FtsI-specific effect rather than a general change in cell envelope physiology [42].

**Fig 5:**
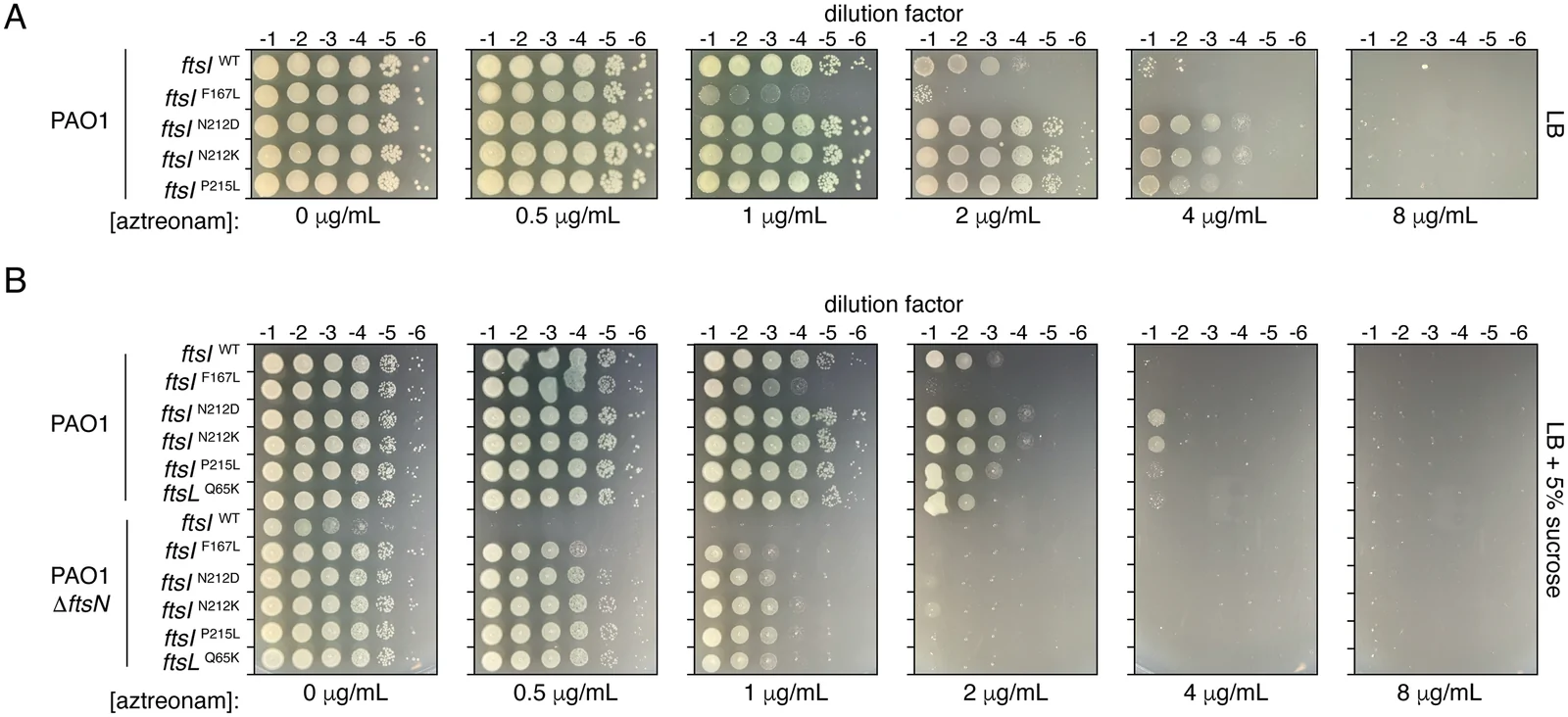
FtsI* variants alter susceptibility to aztreonam. **A)** Tenfold serial dilutions of *P. aeruginosa* PAO1 cells encoding the indicated *ftsI* alleles at the native chromosomal locus were plated on LB agar containing increasing concentrations of aztreonam. **B)** Tenfold serial dilutions of the indicated *P. aeruginosa* PAO1 or *P. aeruginosa* PAO1 ∆*ftsN* strains encoding the indicated *ftsI* alleles at the native chromosomal locus were plated on LB containing 5% (w/v) sucrose and increasing concentrations of aztreonam.

**Fig 6:**
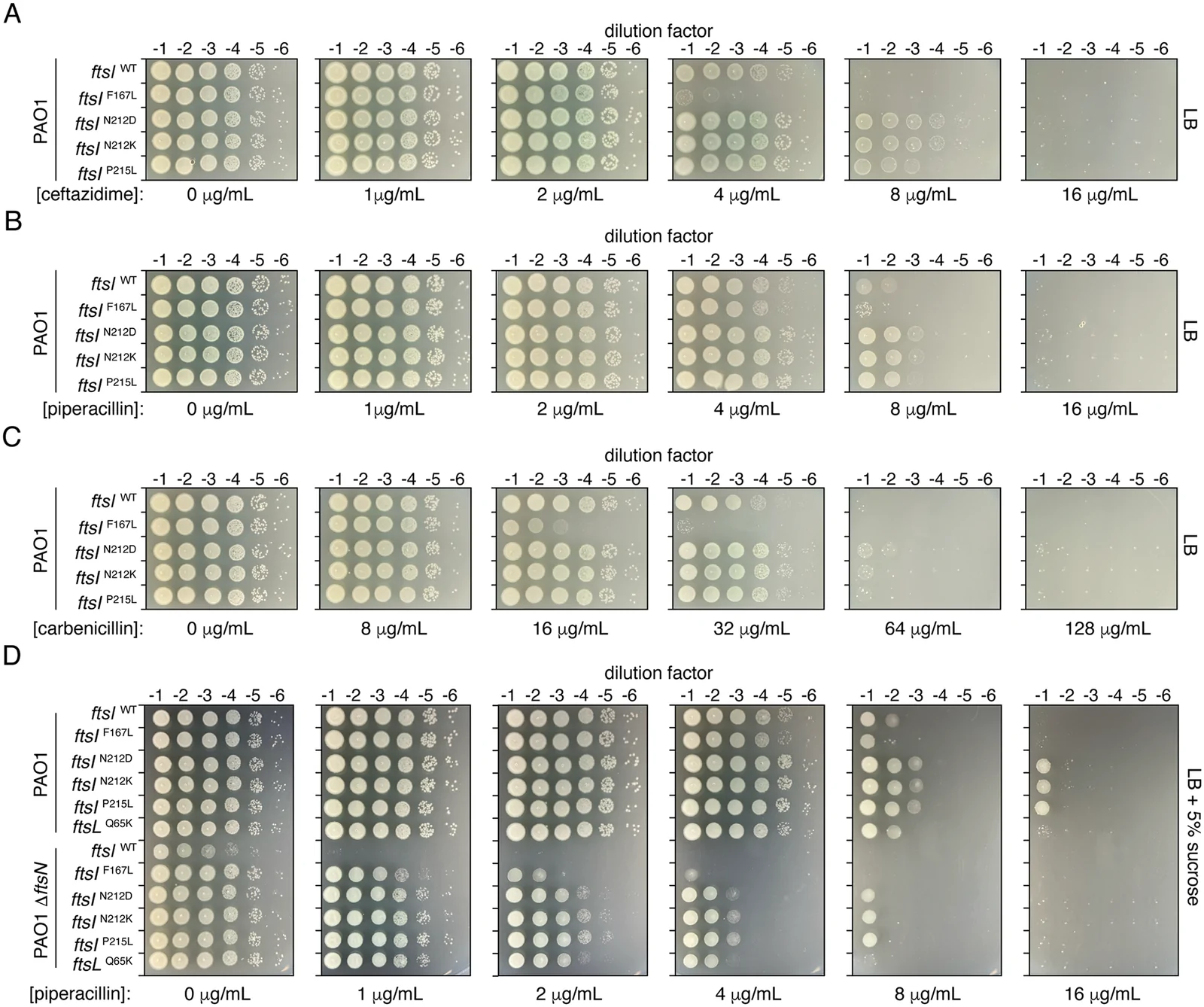
Substitutions in the pedestal domain of FtsI alter susceptibility to divisome-targeting β-lactam antibiotics. A-C). Tenfold serial dilutions of *P. aeruginosa* PAO1 strains encoding the indicated *ftsI* alleles at the native locus were plated on LB agar containing increasing concentrations of the indicated β-lactam antibiotics. **D)** Tenfold serial dilutions of *P. aeruginosa* PAO1 or *P. aeruginosa* PAO1 ∆*ftsN* strains encoding the indicated *ftsI* alleles at the native chromosomal locus were plated on LB agar containing 5% (w/v) sucrose and increasing concentrations of piperacillin.

To ensure that the reduction in aztreonam susceptibility we observed was not due to the contribution of the AmpC β-lactamase, which is constitutively expressed in *P. aeruginosa*, we also examined the growth of our *P. aeruginosa* strains encoding ftsI* in an *ampC* deletion background (S1 Appendix**, Fig E, panel A**). Again, strains carrying *ftsI*^N212D^, *ftsI*^N212K^, or *ftsI*^P215L^ retained their modest reduction in aztreonam susceptibility relative to wild-type. We also performed standardized broth microdilution MIC assays [43], which reproduced the results of our solid media plating assays; wild-type *P. aeruginosa* had an MIC of 4 µg/ml, whereas strains encoding FtsI^N212D^, FtsI^N212K^, or FtsI^P215L^ had an MIC of 8 µg/ml (S1 Appendix**, Fig E, panel B, C**). Together these data indicate that *ampC* is not responsible for the reduced aztreonam susceptibility of our *ftsI* mutants and confirm a twofold increase in aztreonam MIC.

To determine whether the pedestal domain substitutions alter levels of functional FtsI, we used Bocillin-FL, a fluorescent penicillin derivative that labels functional penicillin-binding proteins [42]. Quantification of Bocillin-reactive FtsI revealed comparable levels in strains expressing wild-type FtsI and the pedestal domain variants (S1 Appendix, **Fig F**). These data demonstrate that the reduced β-lactam susceptibility of the pedestal domain variants cannot be explained by altered levels of functional FtsI. Together, these results demonstrate that a subset of FtsN-bypassing substitutions in the FtsI pedestal domain modestly reduce susceptibility to clinically relevant divisome-targeting β-lactams, establishing a link between altered divisome regulation and antibiotic susceptibility.

### FtsI^F167L^ increases susceptibility to divisome-targeting β-lactams

Surprisingly, the *ftsI*^F167L^ allele rendered cells more susceptible to aztreonam than wild type *ftsI* (Fig 5A). Until this point, our genetic data suggested that all FtsI* variants bypass FtsN through a shared mechanism (Figs 1 **and** 4). The finding that *ftsI*^F167L^ confers β-lactam hypersusceptibility contrasts the reduced susceptibility associated with *ftsI*^N212D^, *ftsI*^N212K^, and *ftsI*^P215L^, suggesting that F167L alters FtsWI regulation differently from the other pedestal domain substitutions. Consistent with this idea, F167 lies on the opposite face of the pedestal domain from N212 and P215 and does not participate in the FtsI-FtsL interface. These observations prompted us to investigate the basis for the β-lactam hypersusceptibility associated with *ftsI*^F167L^.

The effectiveness of β-lactams partially stems from their ability to uncouple PG glycosyltransferase and transpeptidase functions, driving a futile cycle of PG polymerization and degradation [31]. We therefore considered whether the hypersusceptibility of the *ftsI*^F167L^ strain depends on FtsN-mediated activation of septal PG synthesis, which could intensify futile cycling in the presence of aztreonam.

If hypersusceptibility depends on FtsN-mediated activation, then removing FtsN should attenuate this phenotype. To test this, we measured aztreonam susceptibility in ∆*ftsN* strains encoding each FtsI* substitution at the native locus. As predicted, the pronounced hypersusceptibility associated with *ftsI*^F167L^ was eliminated in the absence of *ftsN* (Fig 5B). Remarkably, in the ∆*ftsN* background, all four FtsI* variants behaved similarly and conferred reduced aztreonam susceptibility relative to the isogenic ∆*ftsN ftsI*^WT^ strain (Fig 5B). A comparable pattern was observed with piperacillin treatment (Fig 6D), indicating that this phenomenon is not specific to aztreonam. Together, these findings demonstrate that the hypersusceptibility associated with FtsI^F167L^ is FtsN-dependent and distinguish this variant from the resistance-associated pedestal domain substitutions.

## Discussion

Successful bacterial cell division requires that septal PG synthesis be tightly regulated, as any misregulation could have lethal consequences for the cell. It is therefore unsurprising that there are several intricate protein interactions between the essential divisome proteins that control the most important step of this process, PG polymerization by the glycosyltransferase FtsW. Previous work in *P. aeruginosa* has shown that the FtsQLB complex can stimulate the activation of septal PG synthesis through a direct interaction with the PG synthase FtsWI, likely through FtsL (Fig 7A, red arrow) [20,26]. In *E. coli*, FtsQLB associates with both inactive and active FtsWI complexes, with FtsN proposed to promote the active state through interactions with FtsL and FtsI [44]; however, the role of FtsN in *P. aeruginosa* may differ because the essential motif identified in FtsN is not conserved in pseudomonads and FtsN is dispensable in rich media containing sucrose.

**Fig 7:**
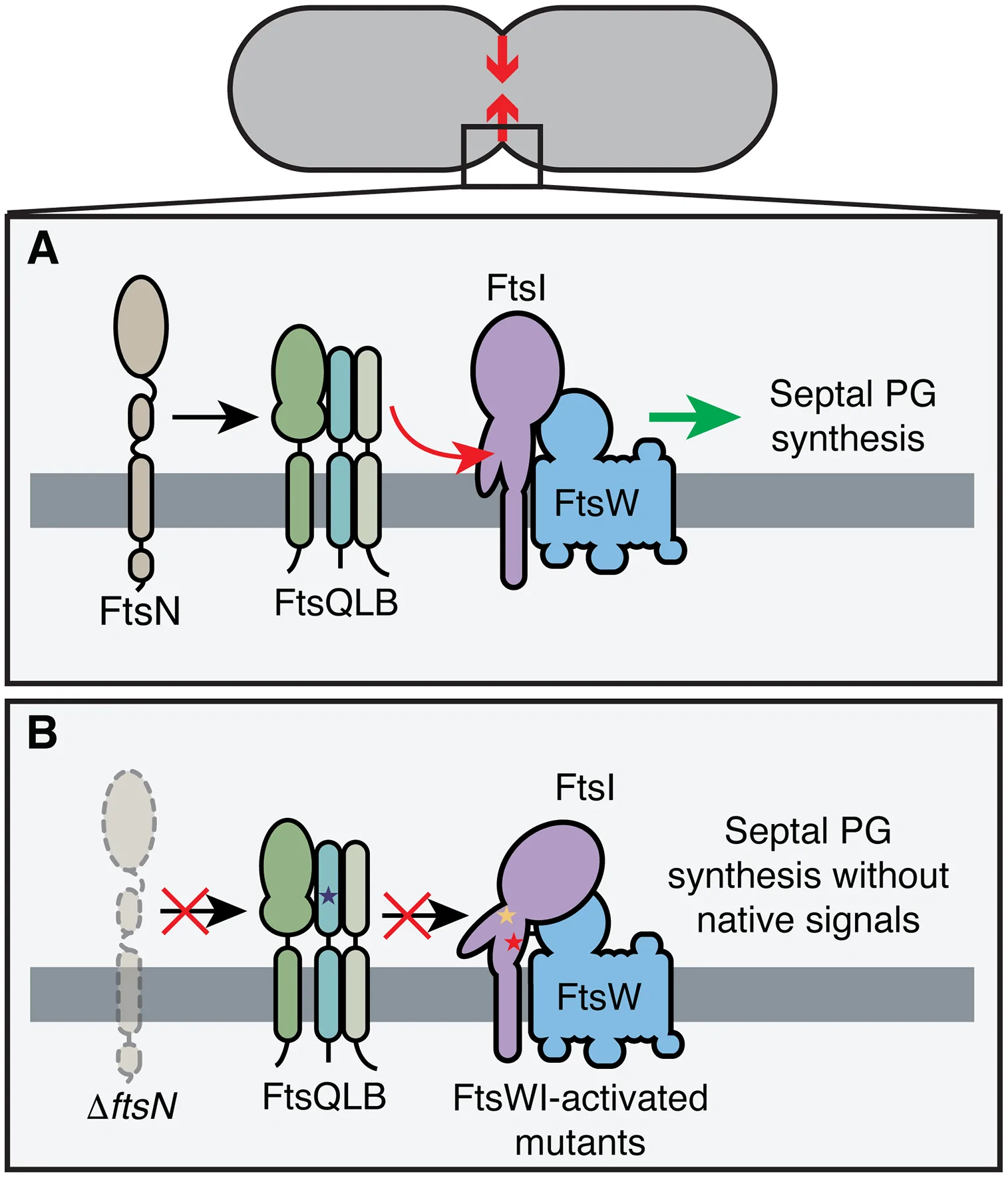
Proposed model of FtsWI regulation by FtsQLB and FtsN. A) FtsN promotes the initiation of cytokinesis by stimulating septal PG synthesis through FtsQLB and the FtsWI synthase. **B)** Substitutions in FtsI (denoted by the red and yellow stars) can bypass the requirement for both FtsN (∆*ftsN*) and FtsQLB-mediated activation (indigo star indicates dominant-negative substitutions in FtsL) *in vivo*. Substitutions at the FtsI-FtsL interface (red star) modestly reduce β-lactam susceptibility, whereas the F167L substitution on the opposite face of the pedestal domain (yellow star) causes FtsN-dependent β-lactam hypersusceptibility.

Here, we identify substitutions in the essential bPBP FtsI that allow FtsWI to function without normal activation by either FtsN or the FtsQLB subcomplex (Fig 7B). All substitutions map to the FtsI pedestal domain, but they fall on two opposing surfaces, supporting the view that this conserved, non-enzymatic region functions as a regulatory hub for FtsWI. Substitutions at the FtsI-FtsL interface restore divisome activity in the absence of upstream signals and modestly reduce susceptibility to several β-lactam antibiotics that preferentially target FtsI (red star, Fig 7B). By contrast, a mutation on the opposite face of the pedestal domain leads to β-lactam hypersusceptibility in an FtsN-dependent manner (yellow star, Fig 7B). Taken together, these distinct phenotypes support a model in which the FtsI pedestal domain integrates multiple regulatory inputs that control septal PG synthesis.

The contrasting drug susceptibilities associated with substitutions on opposing pedestal domain surfaces raise important mechanistic questions. Although our genetic data link altered FtsWI regulation to shifts in β-lactam susceptibility, it is not yet clear how these substitutions influence the futile cycling typically triggered by transpeptidase inhibition [31]. One possibility is that at antibiotic concentrations near the minimal inhibitory concentration (MIC), only a fraction of divisome complexes are inhibited; altered regulation of FtsWI could therefore allow uninhibited complexes to sustain PG synthesis at levels compatible with growth. Another possibility is that the FtsN-bypass mutations subtly rebalance the relationship between FtsW PG glycosyltransferase and FtsI transpeptidase activities, reducing engagement in futile polymerization-degradation cycles and permitting growth at modestly elevated drug concentrations. Understanding how additional divisome components affect the activity of FtsWI during antibiotic exposure may therefore provide insight into the connection between PG synthase activity and β-lactam susceptibility.

The observation that substitutions in the FtsI pedestal domain independently influence β-lactam susceptibility suggests that perturbed regulation of peptidoglycan synthesis may represent a potential contributor to β-lactam resistance. Strikingly, a clinical study by McLean et al. that examined pairs of serially collected *P. aeruginosa* isolates from CF patients treated with inhaled aztreonam identified two isolates encoding *ftsI* substitutions at the same positions identified in our genetic screen. These isolates, encoding *ftsI*^N212T^ or *ftsI*^P215L^ variants, displayed a 256- and 32-fold increase in aztreonam MIC between collection timepoints, respectively. The occurrence of substitutions at these same positions in serial isolates from patients receiving inhaled aztreonam is notable. However, the pedestal domain substitutions identified in our genetic screen produce only a twofold increase in aztreonam MIC when examined in isolation, indicating that they cannot by themselves explain the large changes in susceptibility observed in these clinical isolates. Importantly, these clinical isolates also contain established resistance determinants, including mutations affecting AmpC, OprD, and MexT, as well as additional substitutions within FtsI itself [36]. The isolate containing FtsI N212T also encodes the transpeptidase domain substitution V464A, whereas the isolate containing P215L also encodes R504C, substitutions positioned near the aztreonam binding site that may alter drug-target interactions [45–47]. Thus, pedestal domain substitutions may represent an additional contributor to reduced β-lactam susceptibility, but the relative contribution of these substitutions compared with canonical resistance determinants in clinical isolates remains unresolved. Whether pedestal domain substitutions have additive or synergistic effects with established resistance determinants remains an important question for future study, as does the extent to which pedestal domain substitutions influence susceptibility in other organisms and to antibiotics targeting other bPBPs.

Our findings, together with prior studies of the elongasome PG synthase PBP2, support a conserved role for the pedestal domain as a regulatory element among SEDS-bPBP complexes [5,28]. In the elongasome, gain-of-function mutations in the PBP2 pedestal domain bypass the need for activation by MreC, which inserts directly between the upper and lower lobes of the pedestal domain to promote a hinge-opening motion [5,48]. Although FtsQLB serves as the functional analogue of MreC in the divisome, the structural mechanisms of activation differ because FtsQLB binds along the lateral surface of the FtsI pedestal domain rather than inserting between its lobes [26]. Consistent with this interaction surface, our FtsN-bypassing substitutions cluster at two lateral edges of the pedestal domain rather than at its centre.

Our engineered disulfide-bond experiment, which restricts the FtsI pedestal domain to a closed conformation and produces a dominant-negative phenotype, is consistent with the presence of a hinge-like movement within FtsI’s pedestal domain. How such movement is triggered remains unclear given the lateral mode of FtsQLB binding. Together with the previously described *E. coli* FtsI variant K211I, which lies in the central hinge region, these observations suggest that multiple surfaces of the pedestal domain may serve as receptors for distinct regulatory cues. The distribution of FtsN-bypassing substitutions across multiple pedestal domain surfaces suggests that regulation of the divisome PG synthase may be more complex than that of the elongasome. Defining whether different pedestal domain surfaces interact with upstream regulatory factors, and how these inputs are integrated to coordinate septal PG synthesis with the broader cell division program, will be essential for resolving the mechanistic basis of divisome activation.

More broadly, the ability of regulatory perturbations within the pedestal domain to either potentiate or attenuate β-lactam activity suggests that FtsWI influences antibiotic susceptibility in ways not solely determined by inhibition of transpeptidase activity. Although additional work is needed to establish the underlying mechanisms, a clearer understanding of these processes may help identify points within the activation pathway that could be exploited to enhance the efficacy of existing β-lactams, including aztreonam, in combination therapies.

## Materials and methods

### Bacterial strain and culture conditions

All *P. aeruginosa* strains were derived from the sequenced strain PAO1 [49]. *P. aeruginosa* strains were grown in lysogeny broth (LB, 10 g/L tryptone, 5 g/L NaCl, 5 g/L yeast extract) or VBMM (3.42 g/L trisodium citrate dihydride, 2.0 g/L citric acid, 10 g/L K_2_HPO_4_, 3.5 g/L NaNH_4_PO_4_⋅4H_2_O, 1 mM MgSO_4_, 0.1 mM CaCl_2_) shaking at 220 rpm or on LB, or VBMM containing 1.5% (w/v) agar, as indicated. *P. aeruginosa* was grown at 37°C or 42°C, as indicated. *E. coli* strain XL1 Blue (Novagen) was used for plasmid maintenance and SM10 was used for conjugative transfer. *E. coli* strain LSM9, an *fhuA*^-^ ∆*pbpC*, *∆mtgA* derivative of C43 (DE3), was used for protein expression [20]. The following concentrations of antibiotics were used: ampicillin (Amp), 50 µg/mL; chloramphenicol (Cam), 25 µg/mL; gentamicin (Gent), 15 µg/mL (*E. coli*) or 30 µg/mL (*P. aeruginosa*); kanamycin (Kan), 50 µg/mL. Carbenicillin (BioShop), Aztreonam (AK Scientific), ceftazidime (Sigma), and piperacillin (Sigma) were used at the concentrations indicated.

### Plasmid construction

Plasmids and oligonucleotides used in this study are found in S1 Appendix, tables C and D, respectively. For full details of plasmid construction, please see the supplementary material.

### Allelic replacement in *P. aeruginosa* PAO1

Chromosomal mutations generated in *P. aeruginosa* were constructed using the double allelic exchange method as previously described, with minor modifications [50]. The fusion alleles encoded by pJC9, pJC10, pJC11, and pJC12 were introduced into *P. aeruginosa* via conjugation with the donor strain *E. coli* SM10. Merodiploids were selected at 37°C on VBMM agar containing 30 µg/mL gentamicin. SacB-mediated counterselection was carried out by selecting double cross-over mutations on LB containing 5% (w/v) sucrose. The *ftsI* genes from resulting gentamicin-susceptible colonies were amplified by PCR using oJC20/oJC27 and the resulting amplicons were sequenced by Sanger sequencing to identify clones harbouring the desired mutation. To delete *ftsN*, pJC21 was introduced into *P. aeruginosa* by conjugation with *E. coli* SM10 and by the selection steps described above. Following counterselection, strains containing the desired deletion were identified by PCR using oJC28/oJC30. All ∆*ftsN* strains were maintained on LB containing 5% (w/v) sucrose.

### Electroporation of *P. aeruginosa*

*P. aeruginosa* strains were made competent using previously described methods [51]. 1 mL of stationary-phase *P. aeruginosa* culture was pelleted by centrifugation at 6000xg for 2 minutes before being washed with 1 mL of room temperature 300 mM sucrose. Cells were collected by centrifugation at 6000xg for 2 minutes and washed again with 1 mL 300 mM sucrose. This procedure was repeated for a total of 3 washes with 300 mM sucrose. Cells were resuspended in 50 µL 300 mM sucrose, and 150 ng plasmid DNA suspended in water was added. Cells were transferred to a 1 mm gap electroporation cuvette before electroporation using standard protocols. Transformants were selected by plating on LB or LB containing 5% (w/v) sucrose containing 30 µg/mL gentamicin.

### *P. aeruginosa* viability assays

Overnight cultures of *P. aeruginosa* PAO1 derivatives were normalized to an OD_600_ of 1.0 before being serially diluted. 5 µL aliquots of the dilutions were spotted onto LB or VBMM agar containing the appropriate antibiotics or IPTG, as indicated. Plates were incubated at 37°C for 24 hours before imaging.

### MIC assays

Protocol was adapted from Kadeřábková, N. et al [43]. Briefly, normalized cultures of *P. aeruginosa* were added to 96-well microtitre plates containing aztreonam at the indicated concentrations. Plates were incubated statically at 37^o^C overnight and imaged after 20 hours.

### Purification of *P. aeruginosa* FLAG-FtsW-FtsI variants

*P. aeruginosa* FtsWI complexes were purified as previously described [20]. Briefly, *E. coli* expression strain LSM9 harbouring pAM174 and the appropriate pLSM33-derived expression vectors was grown in 1 L TB supplemented with 2 mM MgCl_2_, kanamycin, and chloramphenicol at 37°C until the OD_600_ was 0.7. The culture was cooled to 20°C before inducing protein expression with 1 mM IPTG and 0.1% (w/v) arabinose. Cells were harvested 18 h post-induction by centrifugation (6000x*g*, 20 mins, 4°C), resuspended in 80 mL buffer B (50 mM HEPES pH 7.5, 150 mM NaCl, 20 mM MgCl_2_, 0.5 mM DTT), and lysed by passage through a cell disruptor (Constant Systems) at 25 000 psi twice. Membranes were collected by ultracentrifugation (100 000xg, 1 h, 4°C). The membrane pellets were resuspended in solubilization buffer B (20 mM HEPES pH 7.0, 0.5 M NaCl, 20% glycerol, 1% (w/v) DDM (ThermoFisher)) and rotated end over end for 1 h at 4°C before ultracentrifugation (100 000xg, 1 h, 4°C) to collect insoluble debris. The supernatant was supplemented with 2 mM CaCl_2_ and loaded onto a homemade M1 α-FLAG antibody resin. The resin was washed with 25 column volumes (CVs) of wash buffer C (20 mM HEPES pH 7.0, 0.5 M NaCl, 20% glycerol, 2 mM CaCl_2_, 0.1% DDM) and the bound protein was eluted from the column with 5 CVs of elution buffer (20 mM HEPES pH 7.0, 0.5 M NaCl, 20% glycerol, 0.1% DDM, 5 mM EDTA pH 8.0, 0.2 mg/mL FLAG peptide). Fractions containing the desired proteins were concentrated and the protein concentration was measured by the Bradford assay. Proteins were aliquoted, snap frozen in liquid nitrogen, and stored at -80°C until use.

### PG glycosyltransferase assays

PG glycosyltransferase assays were performed as previously described, using at least two independent protein purifications [6,20]. Protein concentrations were determined using the Bradford assay by interpolation from a bovine serum albumin standard curve using molecular weights for FtsW-FtsI (108642 g/mol) assuming 1:1 stoichiometry. The indicated protein complex (0.5 µM) was added to a 1x reaction buffer (50 mM HEPES pH 7.0, 10 mM MgCl_2,_ 200 µM cephalexin) containing *E. faecalis* lipid II (10 µM) in a total volume of 10 µL. The samples were incubated at 25°C for the various time points as indicated before being heat-quenched at 95°C for 2 min. After cooling on ice, 4 µL biotin-D-lysine (10 mM) and 0.5 µL *S. aureus* PBP4 (100 µM) were added to the reaction mixture and the samples were incubated for 1 h. Reactions were quenched by the addition of 14.5 µL of 2x Laemmli sample buffer and 10 µL samples were loaded onto a 4–20% SDS polyacrylamide gel (BioRad). The peptidoglycan product was then transferred onto a PVDF membrane using a TransBlot turbo system (BioRad) and the membrane was fixed by incubating in 0.4% paraformaldehyde in phosphate-buffered saline for 30 min. The blot was subsequently blocked using SuperBlock blocking buffer (ThermoFisher) at room temperature for 1 hour. The biotin-labelled products were detected by incubation with IRDye 800CW Streptavidin (1:5000 dilution in SuperBlock buffer). The membrane was washed four times with TBS containing 0.5% Tween-20 before imaging using a ChemiDoc instrument (BioRad).

### Microscopy

Overnight cultures of the indicated *P. aeruginosa* PAO1 or PAO1 ∆*ftsN* strains were diluted 1:500 in the indicated media and grown shaking at 37°C for 4 hours before being immobilized on 1.5% (w/v) M9 agarose pads and covered with #1.5 coverslips. Phase-contrast micrographs were obtained using a Nikon Eclipse Ci-L plus upright microscope equipped with a Nikon Digital Sign Fi3 6MP colour camera, a Plan Apo Lambda x100/1.45 NA oil immersion objective lens and Nikon Elements F acquisition software. For *P. aeruginosa* PAO1 strains expressing FtsL variants under the control of a P_lacUV5_ promoter, overnight cultures were diluted 1:500 in 5 mL LB containing 30 µg/mL gentamicin and grown shaking at 37°C for 2 hours before expression of FtsL variants was induced by the addition of 1 mM IPTG. Cultures were allowed to grow for a further 3 hours shaking at 37°C before cells were fixed in fixing solution (0.4% formaldehyde, 10 mM sodium phosphate pH 7.6). Fixed cells were washed twice with phosphate-buffered saline before being immobilized on 1.5% M9 agarose pads and imaged as described above. Cells were imaged within 24 h of fixation. For quantification analysis of each experiment, at least 150 cells from were analyzed using Omnipose [52].

### Error prone PCR and *ftsI* library generation

Mutagenesis was adapted from a previously described method [53]. Four independent mutant plasmid libraries were constructed by mutagenizing *ftsI* in plasmid pLSM53 using *Taq* polymerase with Thermopol buffer (New England Biolabs, M0267L) and primers oLSM556/oLSM557. The resulting PCR products were purified using the Monarch PCR & DNA cleanup kit (NEB, T1030) and used as ‘megaprimers’ that were denatured and annealed to pLSM53 to amplify the vector backbone using Q5 High-Fidelity polymerase (NEB, M0491S). The reactions were then digested with DpnI to remove parental plasmid DNA and the four libraries were independently electroporated into NEB 10-beta electrocompetent cells (NEB, C3020K) before plating on LB agar containing 15 µg/mL gentamicin at 37°C overnight.

Transformants were resuspended in LB and normalized to an OD_600_ of 10. Cells from 1 mL suspension were centrifuged at 21,000 x *g* for 1 min and plasmid DNA was isolated from the suspension using the Monarch Plasmid DNA miniprep kit (NEB, T1010).

An overnight culture of *E. coli* SM10 was pelleted at 5000 x *g* for 2 min at 4^o^C, and washed three times in 300 mM sucrose to generate electrocompetent cells. All four libraries were independently electroporated into *E. coli* SM10 electrocompetent cells, plated on LB agar containing 15 µg/mL gentamicin, and grown at 37°C overnight.

To introduce the libraries into *P. aeruginosa* PAO1 ∆*ftsN*, lawns of the recipient *P. aeruginosa* strain were plated the night before and allowed to grow at 37°C. The resulting SM10 transformants from each library were collected and independently mixed with cells from the PAO1 ∆*ftsN* lawns. The mixed cells were incubated on an LB agar plate containing 5% (w/v) sucrose at 37°C for 7 hours before being resuspended in LB containing 5% (w/v) sucrose, plated on VBMM containing 5% (w/v) sucrose and 30 µg/mL gentamicin, and grown at 37°C overnight. The resulting PAO1 ∆*ftsN* transformants were collected and resuspended in LB containing 5% (w/v) sucrose and 30 µg/mL gentamicin and each library was normalized to an OD_600_ of 10. 10-fold serial dilutions were plated on VBMM containing 1 mM IPTG and 30 µg/mL gentamicin and grown at 37°C overnight. Individual colonies arising on IPTG-supplemented plates were re-streaked on VBMM with or without IPTG. Those that displayed IPTG dependence were grown overnight in LB containing 5% (w/v) sucrose and 30 µg/mL gentamicin to isolate their plasmids, which were sequenced by Plasmidsaurus using Oxford Nanopore technology. The missense mutations identified in each sequenced plasmid are available in Table A in the S1 Appendix.

### Bocillin-FL labelling of FtsI

Purification of bacterial membranes and labelling of penicillin-binding proteins was adapted from the method of Montaner *et al* [41]. Liquid cultures of PAO1 and *ftsI* derivative strains were grown overnight in LB at 37 °C and then sub-cultured into LB (50 mL) at a dilution of 1:100 until they reached mid-exponential phase (OD_600nm_ = 0.5-0.6). Cultures were harvested by centrifugation (4000 x *g*, 15 min) and washed twice in phosphate-buffered saline. Cell pellets were resuspended in 10 mL of phosphate-buffered saline and lysed using a one-shot cell disruptor (Constant Systems) operating at 30,000 psi. Membranes were collected from lysates by ultracentrifugation (150,000 x *g,* 40 min, 4°C). Crude membrane preparations were resuspended in 500 µL of phosphate-buffered saline using a Dounce homogenizer and total protein in each membrane preparation was quantified by Bradford assay. The crude membrane fractions were normalized to a total protein concentration of 1 mg/mL by dilution with phosphate-buffered saline. Labelling reactions (50 µL) were prepared with 45 µg of total membrane protein and 5 µM Bocillin-FL (Invitrogen, B13233), then incubated in a thermomixer (Eppendorf) for 30 min at 37°C, shaking at 1000 rpm. Reactions were immediately quenched by the addition of 12.5 µL of 4x Laemmli reducing sample buffer. Samples (20 µL) were loaded on a 10% SDS-PAGE gel and separated at 85 V for 20 min followed by 180 V for 50 min. Gels were washed once in deionized water for 5 min and imaged (BioRad ChemiDoc MP) using a 518–546 nm filter. Densitometry of the FtsI (PBP3) band was performed using ImageJ after samples were normalized against the wild-type PBP2 band to control for differences in loading. Results are representative of two independent biological replicates.

### Densitometry analysis

As a measure of glycan polymerization, we measured the signal of individual lanes on each glycosyltransferase assay blot. An area of equal size that encompassed signal for the entire height of each lane, but excluding lipid II, was selected. Intensity measurements were collected from three separate blots. Analysis was performed using ImageJ software.

## Supporting information

S1 Appendix Fig A: Validation of individual FtsI substitutions that bypass FtsN. A) Tenfold serial dilutions of *P. aeruginosa* PAO1 ∆*ftsN* strains harbouring plasmids encoding the indicated *ftsI* alleles under the control of a P_lacUV5_ promoter. Dilutions were plated on LB containing 5% (w/v) sucrose or VBMM containing the indicated concentrations of IPTG to induce expression of the FtsI variants. **Fig B: FtsI pedestal domain substitutions reduce cell length in LB containing 5% sucrose and VBMM.** Cell lengths were quantified from micrographs of *P. aeruginosa* PAO1 cells encoding the indicated *ftsI* alleles at the native chromosomal locus grown in (A) LB containing 5% (w/v) sucrose or (B) VBMM, as shown in Fig 1E. At least 150 cells were measured for each strain. Statistical significance was determined as described in Fig 3 by comparison of each FtsI variant with wild-type FtsI; ***, *p* = 0.0006; ****, *p* < 0.0001. Bars represent the mean. **Fig C: FtsI pedestal domain substitutions suppress the morphology defects caused by activation-defective FtsL variants. A)** Phase contrast micrographs of *P. aeruginosa* PAO1 strains encoding the indicated *ftsI* alleles at the native chromosomal locus and harbouring plasmids encoding the indicated *ftsL* alleles under the control of a P_lacUV5_ promoter. Expression of FtsL variants was induced with 1 mM IPTG after 2 h of growth in LB, and cells were grown for an additional 3 h at 37°C prior to fixation and imaging. Scale bar represents 5 µm. B) Quantification of cell length from micrographs from the experiment shown in (A). Bars represent the mean. Statistical significance was determined as described in Fig 3 by comparison of each FtsI* strain expressing an indicated FtsL variant with the corresponding FtsL variant expressed in the wild-type FtsI background; **** = *p* < 0.0001. **Fig D: Densitometric quantification of PG polymerization assays** Glycan polymer accumulation was quantified by densitometric analysis of the PG glycosyltransferase assays shown in Fig 4D-4E. Data represent the mean of three independent experiments; error bars represent the standard error of the mean. **Fig E: AmpC does not contribute to reduced aztreonam susceptibility of FtsI pedestal domain variants. A)** Tenfold serial dilutions of *P. aeruginosa* PAO1 ∆*ampC* strains encoding the indicated *ftsI* alleles at the native locus were plated on LB agar containing increasing concentrations of aztreonam. Plates were imaged after 24h. **B)** Representative results from a broth microdilution MIC assay. MIC assays were performed in 96-well plates containing the indicated concentrations of aztreonam and incubated statically for 20h at 37^°^C. MIC was defined as the lowest concentration of aztreonam that prevented visible growth. C) Aztreonam MIC values from two independent biological replicates are tabulated. **Fig F: Levels of Bocillin-reactive FtsI are comparable between FtsI pedestal domain variants and wild-type. A)** Bocillin-FL-labelled penicillin-binding proteins from *P. aeruginosa* PAO1 strains encoding the indicated *ftsI* alleles at the native chromosomal locus were separated by SDS-PAGE. **B)** Densitometric quantification of Bocillin-reactive FtsI from two independent biological replicates. PBP2 signal from mutant strains were normalized against PBP2 in the wild-type strain to account for differences in loading, and FtsI signal was adjusted accordingly. Error bars represent the standard deviation. **Table A: Plasmids encoding P**_**lacUV5**_**-*ftsI* variants that restore growth of *P. aeruginosa* ∆*ftsN.* Table B: Strains used in this study. Table C: Plasmids used in this study. Table D: Oligonucleotide primers used in this study.**
(PDF)

S1 File Raw images: PDF file containing all uncropped gel and Western Blots from the figures. (PDF)

S1 Data Excel Spreadsheet containing all numerical data used to generate graphs in the figures. (XLSX)
