# Supplementary material for: Altered regulation of septal peptidoglycan synthesis modulates β-lactam susceptibility in Pseudomonas aeruginosa
Source: PLoS Genet. 2026 Sep 28;22(9):e1012319. doi: 10.1371/journal.pgen.1012319 (PMC13641693; doi:10.1371/journal.pgen.1012319)
Supplement: Supplementary file 1 [file pgen.1012319.s001.pdf]

Supporting Information

Altered Regulation of Septal Peptidoglycan Synthesis Modulates β-Lactam Susceptibility in *Pseudomonas aeruginosa*

Jake Colautti[1,2], Alexander C. Anderson[1,2], Wyatt P. K. Clark[1,2], Lindsey S. Marmont[1,2,*]

[1]Department of Biochemistry and Biomedical Sciences, McMaster University, Hamilton, Ontario, Canada
[2]Michael DeGroote Institute for Infectious Disease Research, McMaster University, Hamilton, Ontario, Canada

* marmontl@mcmaster.ca

Supplementary figures A-F
Supplementary tables A-D
Supplementary references

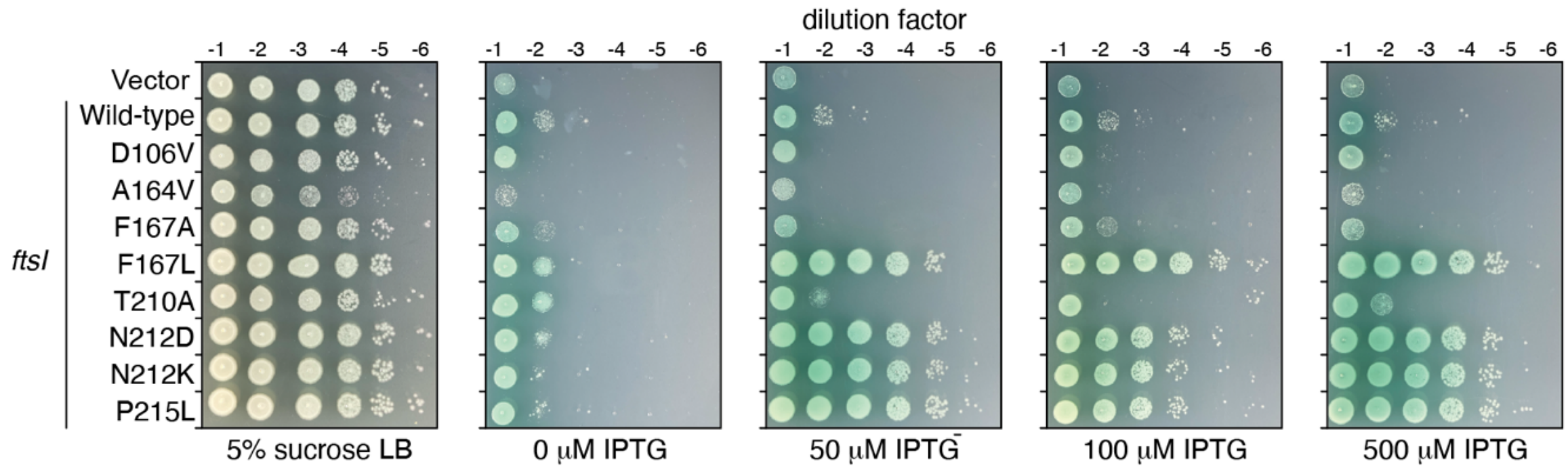

Fig A: Validation of individual FtsI substitutions that bypass FtsN. Tenfold serial dilutions of *P. aeruginosa* PAO1 Δ*ftsN* strains harbouring plasmids encoding the indicated *ftsI* alleles under the control of a P_{lacUV5} promoter. Dilutions were plated on LB containing 5% (w/v) sucrose or VBMM containing the indicated concentrations of IPTG to induce expression of the FtsI variants.

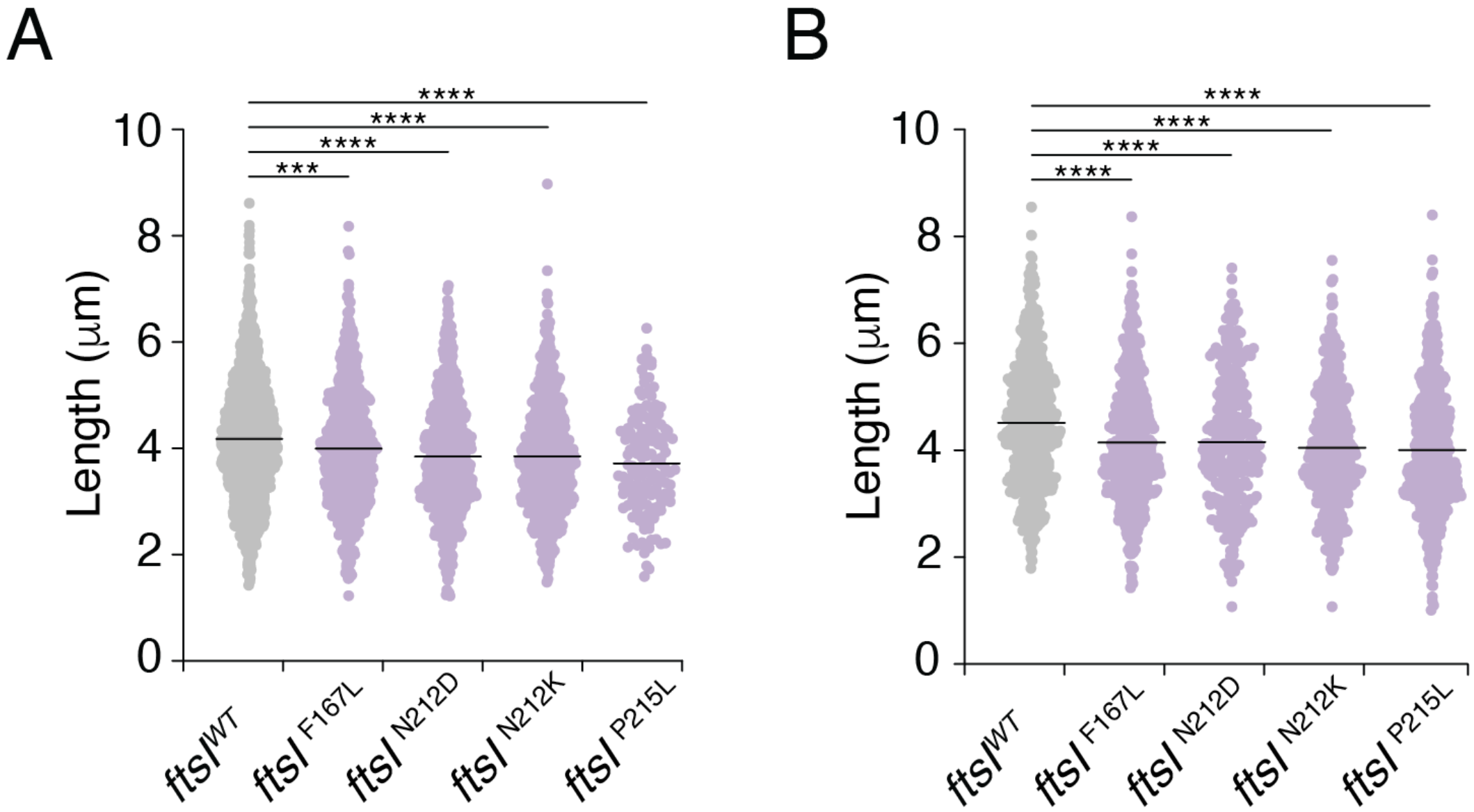

Fig B: FtsI pedestal domain substitutions reduce cell length in LB containing 5% sucrose and VBMM. Cell lengths were quantified from micrographs of *P. aeruginosa* PAO1 cells encoding the indicated *ftsI* alleles at the native chromosomal locus grown in (A) LB containing 5% (w/v) sucrose or (B) VBMM, as shown in Figure 1E. At least 150 cells were measured for each strain. Statistical significance was determined as described in Figure 3 by comparison of each FtsI variant with wild-type FtsI; ***, $p = 0.0006$; ****, $p < 0.0001$. Bars represent the mean.

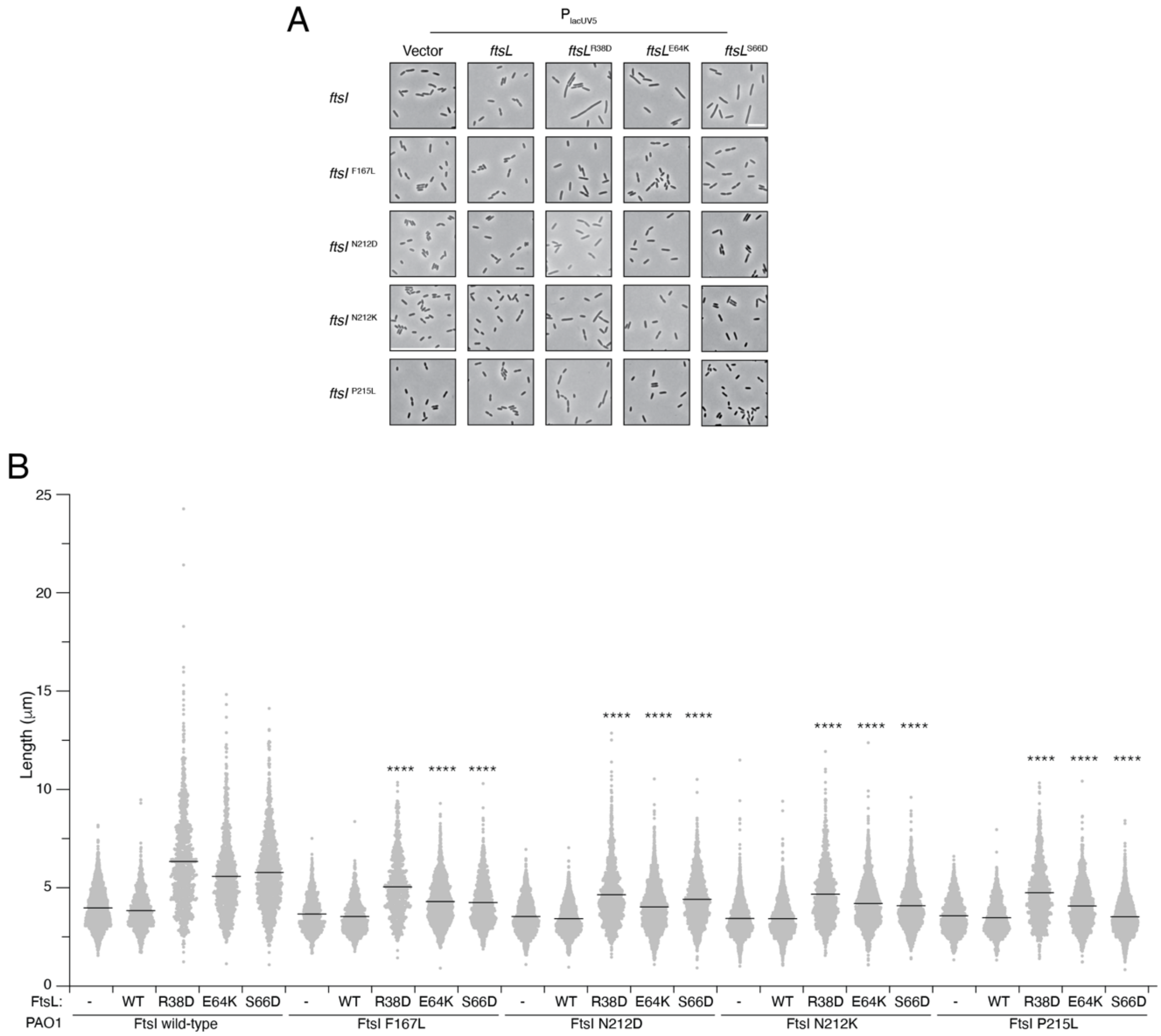

Fig C: FtsI pedestal domain substitutions suppress the morphology defects caused by activation-defective FtsL variants. A) Phase contrast micrographs of *P. aeruginosa* PAO1 strains encoding the indicated *ftsI* alleles at the native chromosomal locus and harbouring plasmids encoding the indicated *ftsL* alleles under the control of a P_{lacUV5} promoter. Expression of FtsL variants was induced with 1 mM IPTG after 2 h of growth in LB, and cells were grown for an additional 3 h at 37°C prior to fixation and imaging. Scale bar represents 5 μm. B) Quantification of cell length from micrographs from the experiment shown in (A). Bars represent the mean. Statistical significance was determined as described in Figure 3 by comparison of each FtsI* strain expressing an indicated FtsL variant with the corresponding FtsL variant expressed in the wild-type FtsI background; **** = $p < 0.0001$.

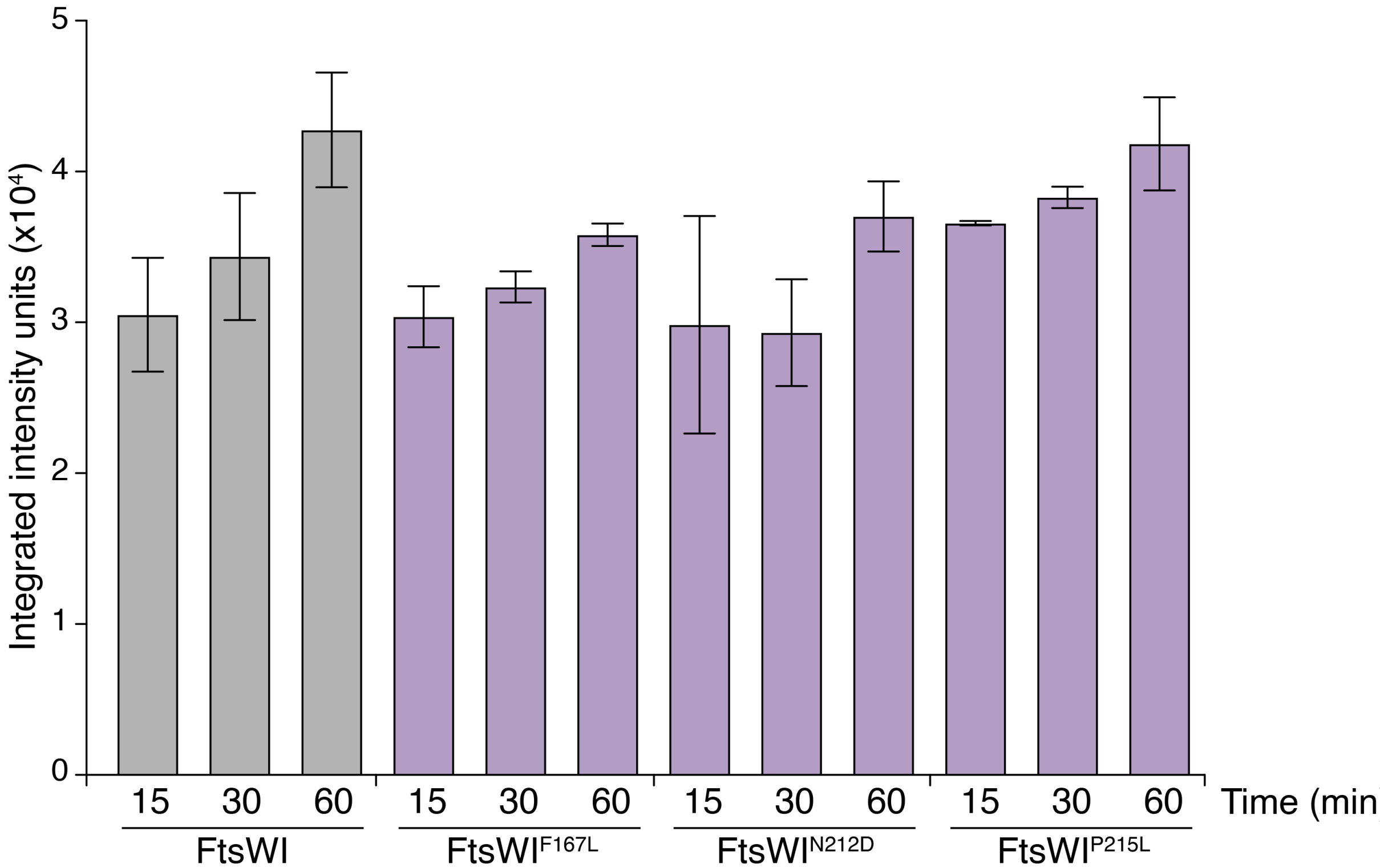

Fig D: Densitometric quantification of PG polymerization assays. Glycan polymer accumulation was quantified by densitometric analysis of the PG glycosyltransferase assays shown in Figure 4D-E. Data represent the mean of three independent experiments; error bars represent the standard error of the mean.

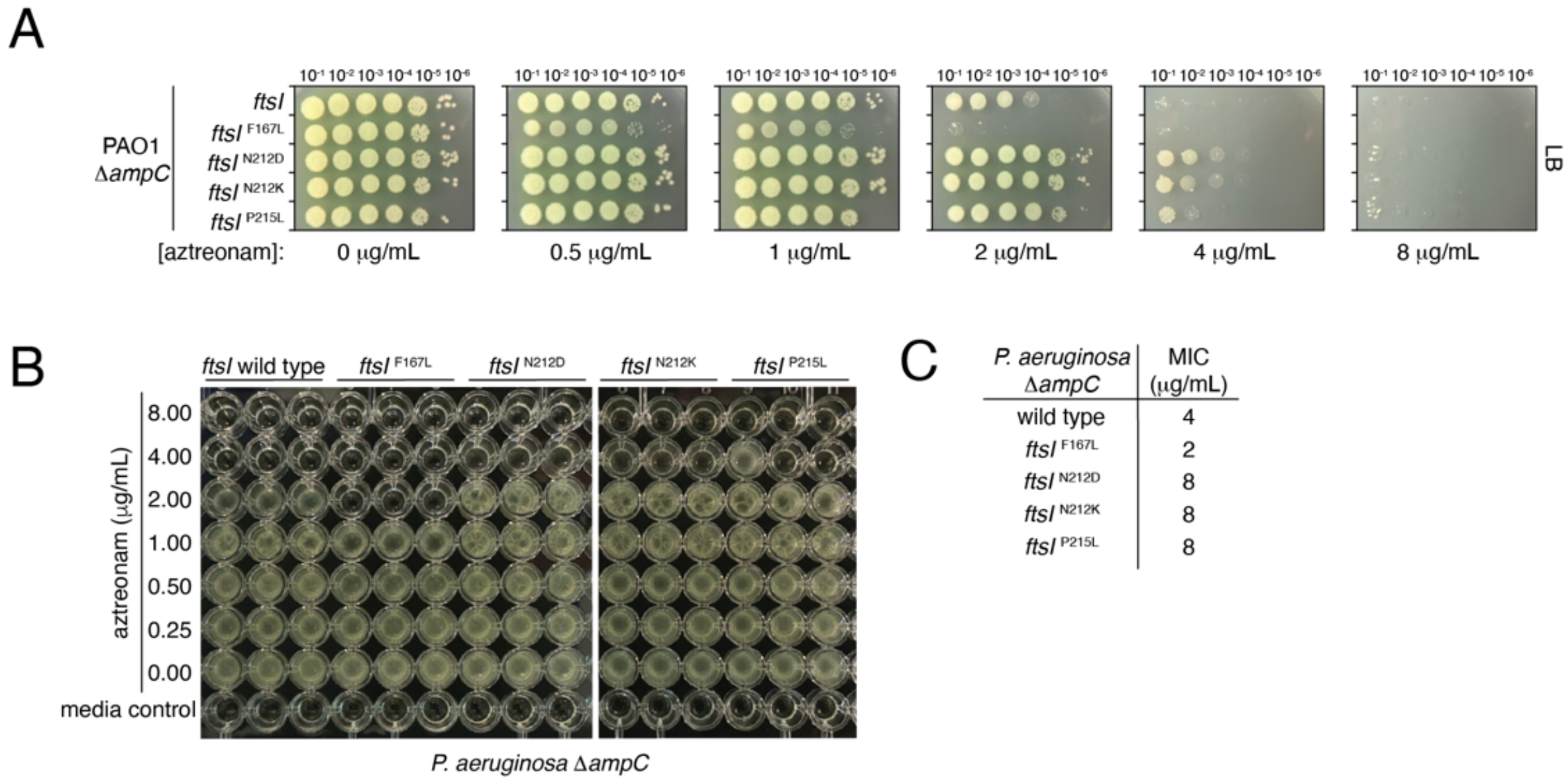

Fig E: AmpC does not contribute to reduced aztreonam susceptibility of FtsI pedestal domain variants. A) Tenfold serial dilutions of *P. aeruginosa* PAO1 Δ*ampC* strains encoding the indicated *ftsI* alleles at the native locus were plated on LB agar containing increasing concentrations of aztreonam. Plates were imaged after 24h. B) Representative results from a broth microdilution MIC assay. MIC assays were performed in 96-well plates containing the indicated concentrations of aztreonam and incubated statically for 20h at 37°C. MIC was defined as the lowest concentration of aztreonam that prevented visible growth. C) Aztreonam MIC values from two independent biological replicates are tabulated.

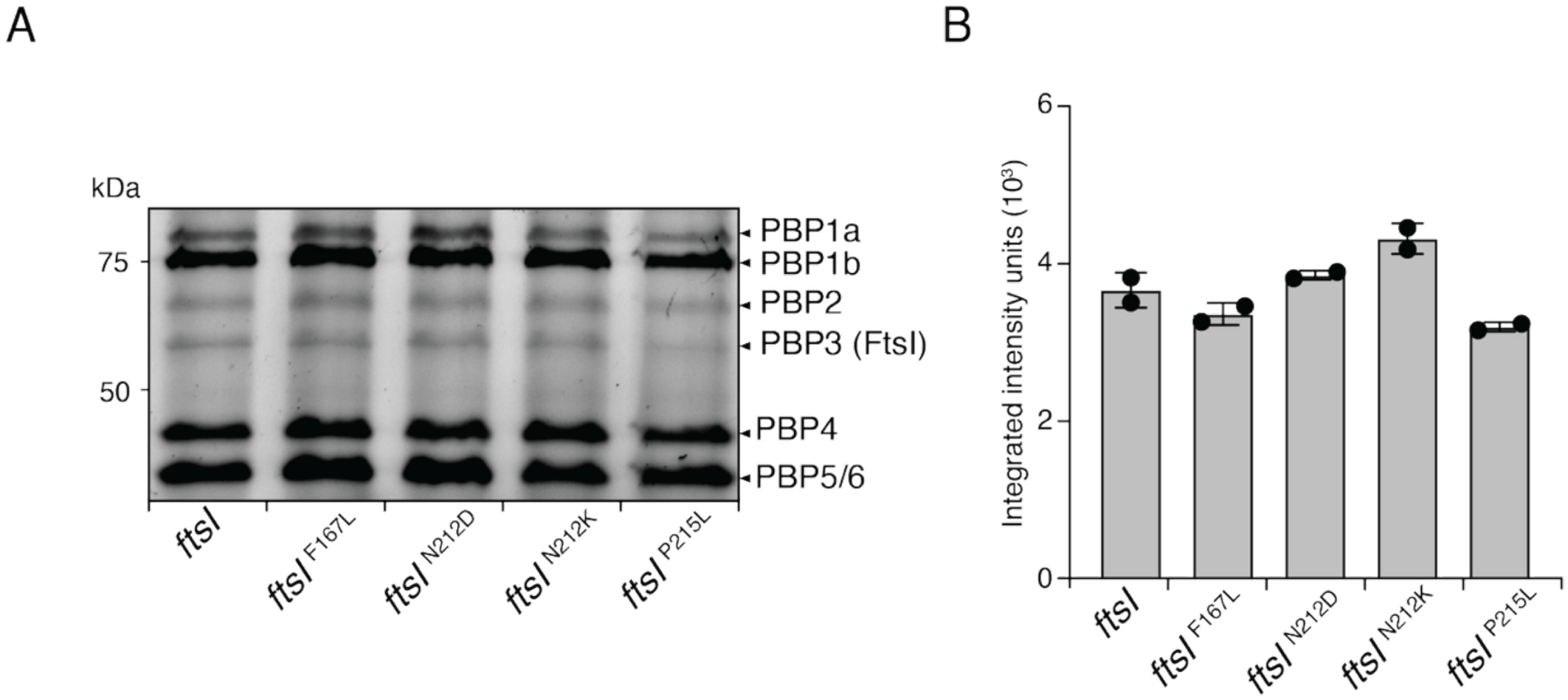

Fig F: Levels of Bocillin-reactive FtsI are comparable between FtsI pedestal domain variants and wild-type. A) Bocillin-FL-labelled penicillin-binding proteins from *P. aeruginosa* PAO1 strains encoding the indicated *ftsI* alleles at the native chromosomal locus were separated by SDS-PAGE. B) Densitometric quantification of Bocillin-reactive FtsI from two independent biological replicates. PBP2 signal from mutant strains were normalized against PBP2 in the wild-type strain to account for differences in loading, and FtsI signal was adjusted accordingly. Error bars represent the standard deviation.

Table A: Plasmids encoding P_{lacUV5}-*ftsI* variants that restore growth of *P. aeruginosa* Δ*ftsN*

**Clone number**	***ftsI* missense mutations[+]**
4-1	I65L, V79A, **F167L**
4-4	D35N, P72L, K529R
4-7	V165I, **N212D**, V442M, R551H
4-9	I65L, V79A, **F167L**,
2-7	**N212K**, I330V
3-9	D106V, **F167L**
3-12	D106V, **F167L**
1-1	V26A, **N212D**
1-4	P72L, A164V
1-7	V26A, **N212D**
1-8	P72L, A164V
1-9	**N212D**
1-11	V26A, **N212D**
2-1	**N212K**
2-3	**F167L**
2-4	P72L, A547T
2-9	**P215L**
2-10	**F167L**
3-2	D106V, **F167L**
3-3	D106V, **F167L**
3-7	D106V, **F167L**
3-8	D106V, **F167L**
3-9	D106V, **F167L**
3-10	D106V, **F167L**
3-11	D106V, **F167L**
4-2	**P215L**
4-4	**F167L**, T210A
4-10	I65L, V79A, **F167L**
4-8	**F167L**, T210A

[+]Bolded alleles indicate the causative substitutions identified in the screen.

Table B: Strains used in this study

Strain	Description	Reference
E. coli		
XL1 Blue	Host strain for plasmid cloning	Novagen
LSM9	C43(DE3) Δ*pbpC* Δ*mtgA fhuA*-	[1]
P. aeruginosa		
PAO1	Wild-type	S. Lory
GT225	PAO1 Δ*ftsN*	[1]
JC1	PAO1 *ftsI*^{F167L}	This study
JC2	PAO1 *ftsI*^{N212D}	This study
JC3	PAO1 *ftsI*^{N212K}	This study
JC4	PAO1 *ftsI*^{P215L}	This study
JC5	PAO1 Δ*ftsN ftsI*^{F167L}	This study
JC6	PAO1 Δ*ftsN ftsI*^{N212D}	This study
JC7	PAO1 Δ*ftsN ftsI*^{N212K}	This study
JC8	PAO1 Δ*ftsN ftsI*^{P215L}	This study
WC1	PAO1Δ*ampC*	This study
WC2	PAO1 Δ*ampC ftsI*^{F167L}	This study
WC3	PAO1 Δ*ampC ftsI*^{N212D}	This study
WC4	PAO1 Δ*ampC ftsI*^{N212K}	This study
WC5	PAO1 Δ*ampC ftsI*^{P215L}	This study

Table C: Plasmids used in this study

Plasmid	Description	Reference
pPSV38	*P. aeruginosa* expression vector containing IPTG-inducible P_{lacUV5} promoter; $Gent^R$	[2]
pEXG2	*P. aeruginosa* allelic exchange vector containing *sacB*; $Gent^R$	[3]
pLSM53	*P. aeruginosa* P_{lacUV5}-*Pa-ftsI* expression vector (pPSV38); $Gent^R$.	[1]
pLSM33	*His6-SUMO-FLAG-Pa-ftsW & Pa-ftsI,* expression vector; Kan^R	[1]
pLSM63	pEXG2 containing *ftsI* fused at the N-terminus to sfGFP, for allelic exchange into *P. aeruginosa*	[1]
pAM174	Encodes arabinose-inducible Ulp1 (L403-K621) protease; Cm^R	[4]
pJC1	*P. aeruginosa* P_{lacUV5}-*Pa-ftsI*^{D106V} expression vector (pPSV38); $Gent^R$.	This study
pJC2	*P. aeruginosa* P_{lacUV5}-*Pa-ftsI*^{A164V} expression vector (pPSV38); $Gent^R$.	This study
pJC3	*P. aeruginosa* P_{lacUV5}-*Pa-ftsI*^{F167A} expression vector (pPSV38); $Gent^R$.	This study
pJC4	*P. aeruginosa* P_{lacUV5}-*Pa-ftsI*^{F167L} expression vector (pPSV38); $Gent^R$.	This study
pJC5	*P. aeruginosa* P_{lacUV5}-*Pa-ftsI*^{T210A} expression vector (pPSV38); $Gent^R$.	This study
pJC6	*P. aeruginosa* P_{lacUV5}-*Pa-ftsI*^{N212D} expression vector (pPSV38); $Gent^R$.	This study
pJC7	*P. aeruginosa* P_{lacUV5}-*Pa-ftsI*^{N212K} expression vector (pPSV38); $Gent^R$.	This study
pJC8	*P. aeruginosa* P_{lacUV5}-*Pa-ftsI*^{P215L} expression vector (pPSV38); $Gent^R$.	This study
pJC9	pEXG2 containing *ftsI* F167L for allelic exchange into *P. aeruginosa*	This study
pJC10	pEXG2 containing *ftsI* N212D for allelic exchange into *P. aeruginosa*	This study
pJC11	pEXG2 containing *ftsI* N212K for allelic exchange into *P. aeruginosa*	This study
pJC12	pEXG2 containing *ftsI* P215L for allelic exchange into *P. aeruginosa*	This study
pLSM11	*P. aeruginosa* P_{lacUV5}-*Pa-ftsL* expression vector (pPSV38); $Gent^R$.	[1]
pLSM79	*P. aeruginosa* P_{lacUV5}-*Pa-ftsL*^{R38D} expression vector (pPSV38); $Gent^R$.	[1]
pLSM83	*P. aeruginosa* P_{lacUV5}-*Pa-ftsL*^{E64K} expression vector (pPSV38); $Gent^R$.	[1]
pLSM84	*P. aeruginosa* P_{lacUV5}-*Pa-ftsL*^{S66D} expression vector (pPSV38); $Gent^R$.	[1]
pLSM91	*P. aeruginosa* P_{lacUV5}-*Pa-ftsI*^{R202C} expression vector (pPSV38); $Gent^R$.	This study
pLSM92	*P. aeruginosa* P_{lacUV5}-*Pa-ftsI*^{Y146C} expression vector (pPSV38); $Gent^R$.	This study
pLSM93	*P. aeruginosa* P_{lacUV5}-*Pa-ftsI*^{E149C} expression vector (pPSV38); $Gent^R$.	This study
pLSM94	*P. aeruginosa* P_{lacUV5}-*Pa-ftsI*^{I58C} expression vector (pPSV38); $Gent^R$.	This study
pLSM95	*P. aeruginosa* P_{lacUV5}-*Pa-ftsI*$^{R202C/Y146C}$ expression vector (pPSV38); $Gent^R$.	This study
pLSM96	*P. aeruginosa* P_{lacUV5}-*Pa-ftsI*$^{E149C/I58C}$ expression vector (pPSV38); $Gent^R$.	This study
pJC21	pEXG2 containing Δ*ftsN* for allelic exchange into *P. aeruginosa*	This study
pJC13	*His6-SUMO-FLAG-Pa-ftsW & Pa-ftsI*^{F167L}*,* expression vector; Kan^R	This study
pJC14	*His6-SUMO-FLAG-Pa-ftsW & Pa-ftsI*^{N212D}*,* expression vector; Kan^R	This study
pJC16	*His6-SUMO-FLAG-Pa-ftsW & Pa-ftsI*^{P215L}*,* expression vector; Kan^R	This study
pWC1	pEXG2 containing Δ*ampC* for allelic exchange into *P. aeruginosa*	This study

Table D: Oligonucleotide primers used in this study

Primer	Sequence (5'-3')
oJC3	GGTGGCCCATGTGGTCGGCTTTA
oJC4	TAAAGCCGACCACATGGGCCACC
oJC5	GCTCGGGCAGGTGACCAAGCTGTTC
oJC6	GAACAGCTTGGTCACCTGCCCGAGC
oJC7	TGCGGTCGGCGCGACCGATGTCG
oJC8	CGACATCGGTCGCGCCGACCGCA
oJC9	CGACATCGGTTAACCCGAC
oJC10	GTCGGGTTAACCGATGTCG
oJC11	GGTCACCAAGGATGCCAAACCGG
oJC12	CCGGTTTGGCATCCTTGGTGACC
oJC13	GGTCACCAAGAAAGCCAAACCGG
oJC14	CCGGTTTGGCTTTCTTGGTGACC
oJC15	GAATGCCAAACTGGGCAAGACCC
oJC16	GGGTCTTGCCCAGTTTGGCATTC
oJC17	CGTGCAGGTCGCGAAGAATGCCA
oJC18	TGGCATTCTTCGCGACCTGCACG
oJC19	ATGTAAAGCAAGCTTGACTCTCCCACTTCTTCCCACTC
oJC20	AAGGTACCGAATTCTCAGCCACGCCCTCCTTTTGC
oJC21	TATTAGTTAAGTATAAGAAGGAGATATACATATATGAAACTGAATTATTTCCAGGGCGCC
oJC22	GCAGCAGCCTAGGTTAATCAGCCACGCCCTCCTTTTGC
oJC23	TGATTAACCTAGGCTGCTG
oJC24	ATATGTATATCTCCTTCTTATACTTAAC
oJC28	TGTAAAGCAAGCTTCATGGAGCTGGAGCAC
oJC29	AAGGTACCGAATTCGTCATGGACATTCGTTTACTC
oJC30	ATGTTCTTCGGTGTTACCTCGG
oLSM556	CCAGGCTTTACACTTTATGCTTCCG
oLSM557	CTGCGCAACTGTTGGGAAGG
oLSM227	GATCGCCGTGGCTGCGTGATCAAGGAC
oLSM228	GTCCTTGATCACGCAGCCACGGCGATC
oLSM229	GTGCCCGGCGTGTGCTCCATCGAGGAG
oLSM230	CTCCTCGATGGAGCACACGCCGGGCAC
oLSM231	GTGTACTCCATCTGCGAGTTTCGGCGT
oLSM232	ACGCCGAAACTCGCAGATGGAGTACAC
oLSM233	CGGCATATCGCCTGCCCTGCGCACCGG
oLSM234	CCGGTGCGCAGGGCAGGCGATATGCCG
oJC33	AGGTCGACTCTAGATTGTTGTGGGTGGACAGGC
oJC34	GCGCTTCAGCATGAGGATTGGCGTCCTTTG
oJC35	CCTCATGCTGAAGCGCTGAAGCGCG
oJC36	TTAATTAAGGTACCAATAACGCCATGGGCGTTATC
oJC37	AAGACGATGCTCCGGGTCAGT
oJC38	ATACCATCAAGGGCAGCGCTT

*Restriction site underlined

Plasmid construction

pJC1 [P_{lacUV5}::^{Pa}*ftsI*^{D106V}(PA4418)] is a pPSV38 derivative

pPSV38 was digested with SacI/XbaI to generate the plasmid backbone. The 5' fragment of *P. aeruginosa ftsI* was PCR amplified from pLSM53 using oLSM556/oJC6, and the 3' fragment of *P. aeruginosa ftsI* was amplified from pLSM53 using oJC5/oLSM557. The two fragments were joined together by overlap extension PCR using oLSM556/oLSM557. After digestion with SacI/XbaI, the PCR product was ligated into pPSV38 to generate pJC1. The final construct was sequence verified by Plasmidsaurus using Oxford Nanopore technology. The *ftsI* gene in this and all related constructs below is inducible with IPTG.

pJC2 [P_{lacUV5}::^{Pa}*ftsI*^{A164V}(PA4418)] is a pPSV38 derivative

pPSV38 was digested with SacI/XbaI to generate the plasmid backbone. The 5' fragment of *P. aeruginosa ftsI* was PCR amplified from pLSM53 using oLSM556/oJC4, and the 3' fragment of *P. aeruginosa ftsI* was amplified from pLSM53 using oJC3/oLSM557. The two fragments were joined together by overlap extension PCR using oLSM556/oLSM557. After digestion with SacI/XbaI, the PCR product was ligated into pPSV38 to generate pJC2. The final construct was sequence verified by Plasmidsaurus using Oxford Nanopore technology.

pJC3 [P_{lacUV5}::^{Pa}*ftsI*^{F167A}(PA4418)] is a pPSV38 derivative

pPSV38 was digested with SacI/XbaI to generate the plasmid backbone. The 5' fragment of *P. aeruginosa ftsI* was PCR amplified from pLSM53 using oLSM556/oJC8, and the 3' fragment of *P. aeruginosa ftsI* was amplified from pLSM53 using oJC7/oLSM557. The two fragments were joined together by overlap extension PCR using oLSM556/oLSM557. After digestion with SacI/XbaI, the PCR product was ligated into pPSV38 to generate pJC3. The final construct was sequence verified by Plasmidsaurus using Oxford Nanopore technology.

pJC4 [P_{lacUV5}::^{Pa}*ftsI*^{F167L}(PA4418)] is a pPSV38 derivative

pPSV38 was digested with SacI/XbaI to generate the plasmid backbone. The 5' fragment of *P. aeruginosa ftsI* was PCR amplified from pLSM53 using oLSM556/oJC10, and the 3' fragment of *P. aeruginosa ftsI* was amplified from pLSM53 using oJC9/oLSM557. The two fragments were joined together by overlap extension PCR using oLSM556/oLSM557. After digestion with SacI/XbaI, the PCR product was ligated into pPSV38 to generate pJC4. The final construct was sequence verified by Plasmidsaurus using Oxford Nanopore technology.

pJC5 [P_{lacUV5}::^{Pa}*ftsI*^{T210A}(PA4418)] is a pPSV38 derivative

pPSV38 was digested with SacI/XbaI to generate the plasmid backbone. The 5' fragment of *P. aeruginosa ftsI* was PCR amplified from pLSM53 using oLSM556/oJC18, and the 3' fragment of *P. aeruginosa ftsI* was amplified from pLSM53 using oJC17/oLSM557. The two fragments were joined together by overlap extension PCR using oLSM556/oLSM557. After digestion with SacI/XbaI, the PCR product was ligated into pPSV38 to generate pJC5. The final construct was sequence verified by Plasmidsaurus using Oxford Nanopore technology.

pJC6 [P_{lacUV5}::^{Pa}*ftsI*^{N212D}(PA4418)] is a pPSV38 derivative

pPSV38 was digested with SacI/XbaI to generate the plasmid backbone. The 5' fragment of *P. aeruginosa ftsI* was PCR amplified from pLSM53 using oLSM556/oJC12, and the 3' fragment of *P. aeruginosa ftsI* was amplified from pLSM53 using oJC11/oLSM557. The two fragments were joined together by overlap extension PCR using oLSM556/oLSM557. After digestion with

SacI/XbaI, the PCR product was ligated into pPSV38 to generate pJC6. The final construct was sequence verified by Plasmidsaurus using Oxford Nanopore technology.

pJC7 [P_{lacUV5}::^{Pa}*ftsI*^{N212K}(PA4418)] is a pPSV38 derivative

pPSV38 was digested with SacI/XbaI to generate the plasmid backbone. The 5' fragment of *P. aeruginosa ftsI* was PCR amplified from pLSM53 using oLSM556/oJC14, and the 3' fragment of *P. aeruginosa ftsI* was amplified from pLSM53 using oJC13/oLSM557. The two fragments were joined together by overlap extension PCR using oLSM556/oLSM557. After digestion with SacI/XbaI, the PCR product was ligated into pPSV38 to generate pJC7. The final construct was sequence verified by Plasmidsaurus using Oxford Nanopore technology.

pJC8 [P_{lacUV5}::^{Pa}*ftsI*^{P215L}(PA4418)] is a pPSV38 derivative

pPSV38 was digested with SacI/XbaI to generate the plasmid backbone. The 5' fragment of *P. aeruginosa ftsI* was PCR amplified from pLSM53 using oLSM556/oJC16, and the 3' fragment of *P. aeruginosa ftsI* was amplified from pLSM53 using oJC15/oLSM557. The two fragments were joined together by overlap extension PCR using oLSM556/oLSM557. After digestion with SacI/XbaI, the PCR product was ligated into pPSV38 to generate pJC8. The final construct was sequence verified by Plasmidsaurus using Oxford Nanopore technology.

pJC9 [*ftsI*^{F167L}(PA4418)] is a pEXG2 derivative

pEXG2 was digested with EcoRI/HindIII to generate the plasmid backbone. To generate an allelic exchange plasmid containing *ftsI*^{F167L}, the homologous region upstream of *ftsI* including the promoter for the operon was PCR amplified from PAO1 genomic DNA using oJC19/oJC10. The homologous region downstream of the desired mutation was amplified using oJC9/oJC20, and the two homologous regions were joined together by overlap extension PCR using oJC19/oJC20. After digestion with EcoRI/HindIII, the PCR product was ligated into pEXG2 to generate pJC9. The final construct was sequence verified by Plasmidsaurus using Oxford Nanopore technology.

pJC10 [*ftsI*^{N212D}(PA4418)] is a pEXG2 derivative

pEXG2 was digested with EcoRI/HindIII to generate the plasmid backbone. To generate an allelic exchange plasmid containing *ftsI*^{N212D}, the homologous region upstream of *ftsI* including the promoter for the operon was PCR amplified from PAO1 genomic DNA using oJC19/oJC12. The homologous region downstream of the desired mutation was amplified using oJC11/oJC20, and the two homologous regions were joined together by overlap extension PCR using oJC19/oJC20. After digestion with EcoRI/HindIII, the PCR product was ligated into pEXG2 to generate pJC10. The final construct was sequence verified by Plasmidsaurus using Oxford Nanopore technology.

pJC11 [*ftsI*^{N212K}(PA4418)] is a pEXG2 derivative

pEXG2 was digested with EcoRI/HindIII to generate the plasmid backbone. To generate an allelic exchange plasmid containing *ftsI*^{N212K}, the homologous region upstream of *ftsI* including the promoter for the operon was PCR amplified from PAO1 genomic DNA using oJC19/oJC14. The homologous region downstream of the desired mutation was amplified using oJC13/oJC20, and the two homologous regions were joined together by overlap extension PCR using oJC19/oJC20. After digestion with EcoRI/HindIII, the PCR product was ligated into pEXG2 to generate pJC11. The final construct was sequence verified by Plasmidsaurus using Oxford Nanopore technology.

pJC12 [*ftsI*^{P215L}(PA4418)] is a pEXG2 derivative

pEXG2 was digested with EcoRI/HindIII to generate the plasmid backbone. To generate an allelic exchange plasmid containing *ftsI*^{P215L}, the homologous region upstream of *ftsI* including the

promoter for the operon was PCR amplified from PAO1 genomic DNA using oJC19/oJC16. The homologous region downstream of the desired mutation was amplified using oJC15/oJC20, and the two homologous regions were joined together by overlap extension PCR using oJC19/oJC20. After digestion with EcoRI/HindIII, the PCR product was ligated into pEXG2 to generate pJC12. The final construct was sequence verified by Plasmidsaurus using Oxford Nanopore technology.

pJC21 [Δ*ftsN* (PA5052)] is a pEXG2 derivative

pEXG2 was digested with EcoRI/HindIII to generate the plasmid backbone. To generate an allelic exchange plasmid with the deleted *ftsN* gene, the homologous region lacking *ftsN* was amplified from *P. aeruginosa* strain GT225 genomic DNA using primers oJC28/oJC29 [1]. After digestion with EcoRI/HindIII, the PCR product was ligated into pEXG2 to generate pJC21. The final construct was sequence verified by Plasmidsaurus using Oxford Nanopore technology.

pJC13 [P_{T7}::H-SUMO-FLAG-^{Pa}*ftsW*(PA4413) and P_{T7}::^{Pa}*ftsI*^{F167L}(PA4418)] is a pLSM33 derivative

pLSM33 was amplified by PCR using oJC23/oJC24 to generate the backbone. The 5' fragment of *P. aeruginosa ftsI* was PCR amplified from pLSM33 using oJC10/oJC21 and the 3' fragment was PCR amplified using oJC9/oJC22. The resulting fragments were joined by overlap extension PCR and assembled into the amplified pLSM33 backbone using isothermal assembly to generate pJC13.

pJC14 [P_{T7}::H-SUMO-FLAG-^{Pa}*ftsW*(PA4413) and P_{T7}::^{Pa}*ftsI*^{N212D}(PA4418)] is a pLSM33 derivative

pLSM33 was amplified by PCR using oJC23/oJC24 to generate the backbone. The 5' fragment of *P. aeruginosa ftsI* was PCR amplified from pLSM33 using oJC12/oJC21 and the 3' fragment was PCR amplified using oJC11/oJC22. The resulting fragments were joined by overlap extension PCR and assembled into the amplified pLSM33 backbone using isothermal assembly to generate pJC14.

pJC15 [P_{T7}::H-SUMO-FLAG-^{Pa}*ftsW*(PA4413) and P_{T7}::^{Pa}*ftsI*^{N212K}(PA4418)] is a pLSM33 derivative

pLSM33 was amplified by PCR using oJC23/oJC24 to generate the backbone. The 5' fragment of *P. aeruginosa ftsI* was PCR amplified from pLSM33 using oJC14/oJC21 and the 3' fragment was PCR amplified using oJC13/oJC22. The resulting fragments were joined by overlap extension PCR and assembled into the amplified pLSM33 backbone using isothermal assembly to generate pJC15.

pJC16 [P_{T7}::H-SUMO-FLAG-^{Pa}*ftsW*(PA4413) and P_{T7}::^{Pa}*ftsI*^{P215L}(PA4418)] is a pLSM33 derivative

pLSM33 was amplified by PCR using oJC23/oJC24 to generate the backbone. The 5' fragment of *P. aeruginosa ftsI* was PCR amplified from pLSM33 using oJC16/oJC21 and the 3' fragment was PCR amplified using oJC15/oJC22. The resulting fragments were joined by overlap extension PCR and assembled into the amplified pLSM33 backbone using isothermal assembly to generate pJC16.

pLSM91 [P_{lacUV5}::^{Pa}*ftsI*^{R202C}(PA4418)] is a pPSV38 derivative

Using pLSM53 as a template, R202 was mutated to C using site-directed mutagenesis (QuikChange Lightning, Agilent) using the primers oLSM227 and oLSM228. The final construct was sequence verified using primers oLSM556 and oLSM557.

pLSM92 [P_{lacUV5}::^{Pa}*ftsI*^{Y146C}(PA4418)] is a pPSV38 derivative

Using pLSM53 as a template, Y146 was mutated to C using site-directed mutagenesis (QuikChange Lightning, Agilent) using the primers oLSM229 and oLSM230. The final construct was sequence verified using primers oLSM556 and oLSM557.

pLSM93 [P_{lacUV5}::^{Pa}*ftsI*^{E149C}(PA4418)] is a pPSV38 derivative

Using pLSM53 as a template, E149 was mutated to C using site-directed mutagenesis (QuikChange Lightning, Agilent) using the primers oLSM231 and oLSM232. The final construct was sequence verified using primers oLSM556 and oLSM557.

pLSM94 [P_{lacUV5}::^{Pa}*ftsI*^{I58C}(PA4418)] is a pPSV38 derivative

Using pLSM53 as a template, I58 was mutated to C using site-directed mutagenesis (QuikChange Lightning, Agilent) using the primers oLSM233 and oLSM234. The final construct was sequence verified using primers oLSM556 and oLSM557.

pLSM95 [P_{lacUV5}::^{Pa}*ftsI*$^{R202C/Y146C}$ (PA4418)] is a pPSV38 derivative

Using pLSM91 as a template, Y146 was mutated to C using site-directed mutagenesis (QuikChange Lightning, Agilent) using the primers oLSM229 and oLSM230. The final construct was sequence verified using primers oLSM556 and oLSM557.

pLSM96 [P_{lacUV5}::^{Pa}*ftsI*$^{E149C/I58C}$(PA4418)] is a pPSV38 derivative

Using pLSM93 as a template, I58 was mutated to C using site-directed mutagenesis (QuikChange Lightning, Agilent) using the primers oLSM233 and oLSM234. The final construct was sequence verified using primers oLSM556 and oLSM557.

pWC1 [Δ*ampC* (PA4110)] is a pEXG2 derivative

To generate an allelic exchange plasmid with the deleted *ampC* gene, 546 bp of the sequence upstream and sequence encoding the first residue of *ampC* was amplified using oJC33 and oJC34. 559 bp of the downstream sequence, including the last three residues and the stop codon of *ampC* were amplified using oJC35 and oJC36. PCR products were joined using overlap extension PCR using the primers oJC33 and oJC36. Following digestion with XbaI and KpnI, the insert was ligated into pEXG2 and sequence verified by Plasmidsaurus using Oxford Nanopore technology.

Supplementary references

1 Marmont, L. S. & Bernhardt, T. G. A conserved subcomplex within the bacterial cytokinetic ring activates cell wall synthesis by the FtsW-FtsI synthase. *Proc Natl Acad Sci U S A* **117**, 23879–23885 (2020). https://doi.org/10.1073/pnas.2004598117

2 Vvedenskaya, I. O. *et al.* Growth phase-dependent control of transcription start site selection and gene expression by nanoRNAs. *Genes Dev* **26**, 1498–1507 (2012). https://doi.org/10.1101/gad.192732.112

3 Hmelo, L. R. *et al.* Precision-engineering the Pseudomonas aeruginosa genome with two-step allelic exchange. *Nat Protoc* **10**, 1820–1841 (2015). https://doi.org/10.1038/nprot.2015.115

4 Meeske, A. J. *et al.* SEDS proteins are a widespread family of bacterial cell wall polymerases. *Nature* **537**, 634–638 (2016). https://doi.org/10.1038/nature19331
