# Supplementary material for: Altered regulation of septal peptidoglycan synthesis modulates β-lactam susceptibility in Pseudomonas aeruginosa
Source: PLoS Genet. 2026 Sep 28;22(9):e1012319. doi: 10.1371/journal.pgen.1012319 (PMC13641693; doi:10.1371/journal.pgen.1012319)

Figure 4C: Captured using BioRad Chemidoc, colorimetric

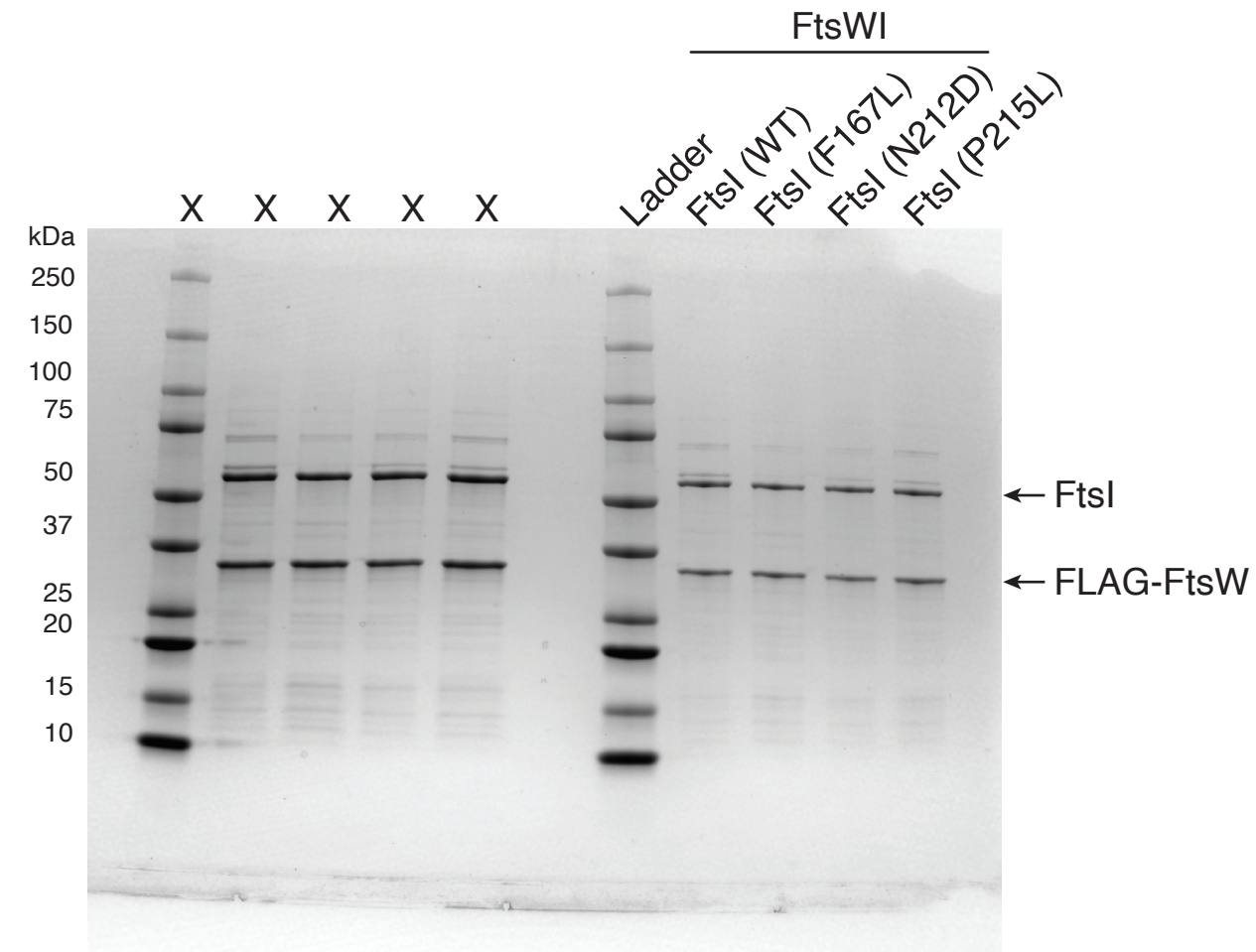

Figure 4D: Captured using BioRad Chemidoc, Chemiluminescence

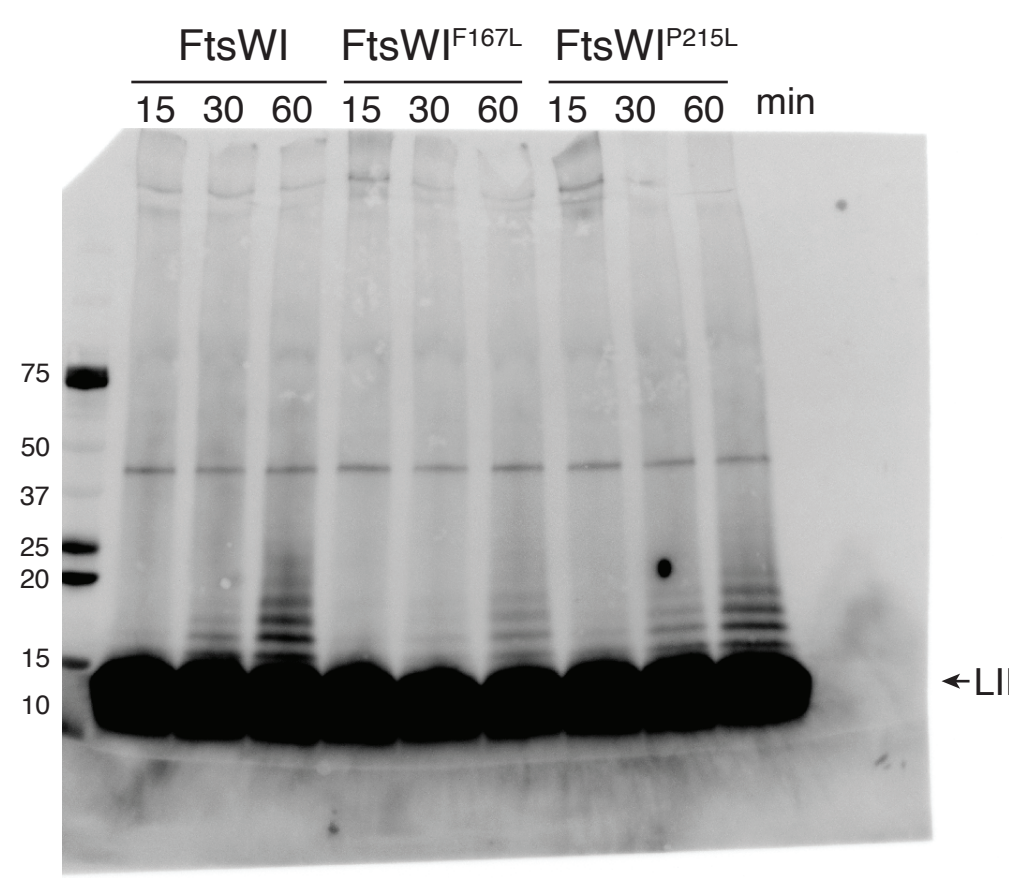

Figure 4E: Captured using BioRad Chemidoc, Chemiluminescence

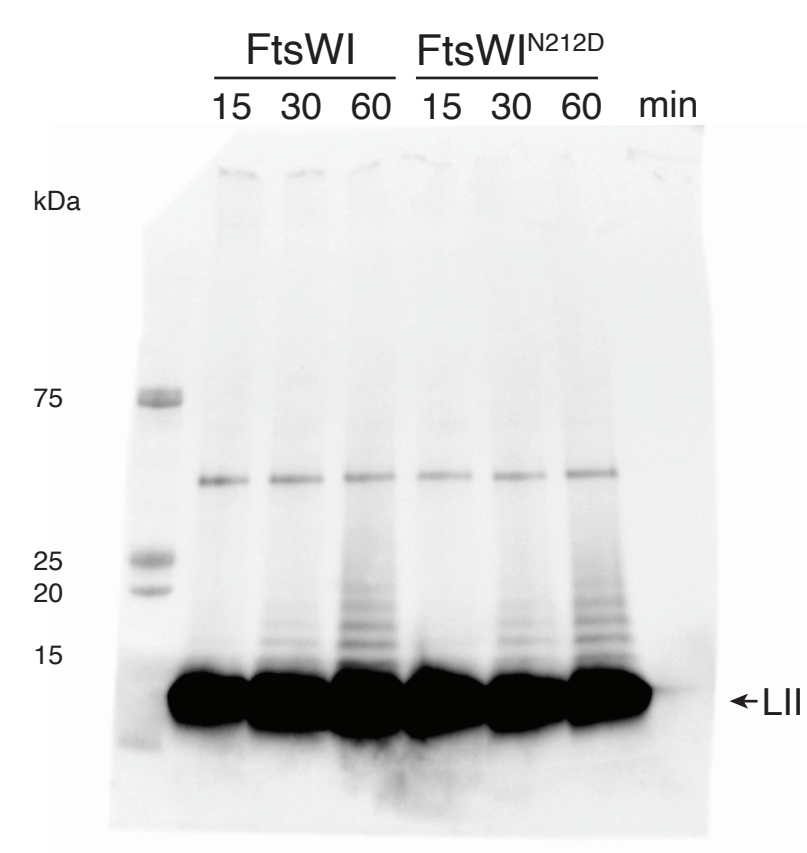

Figure S6A: Captured using BioRad Chemidoc, 518-546 nm filter

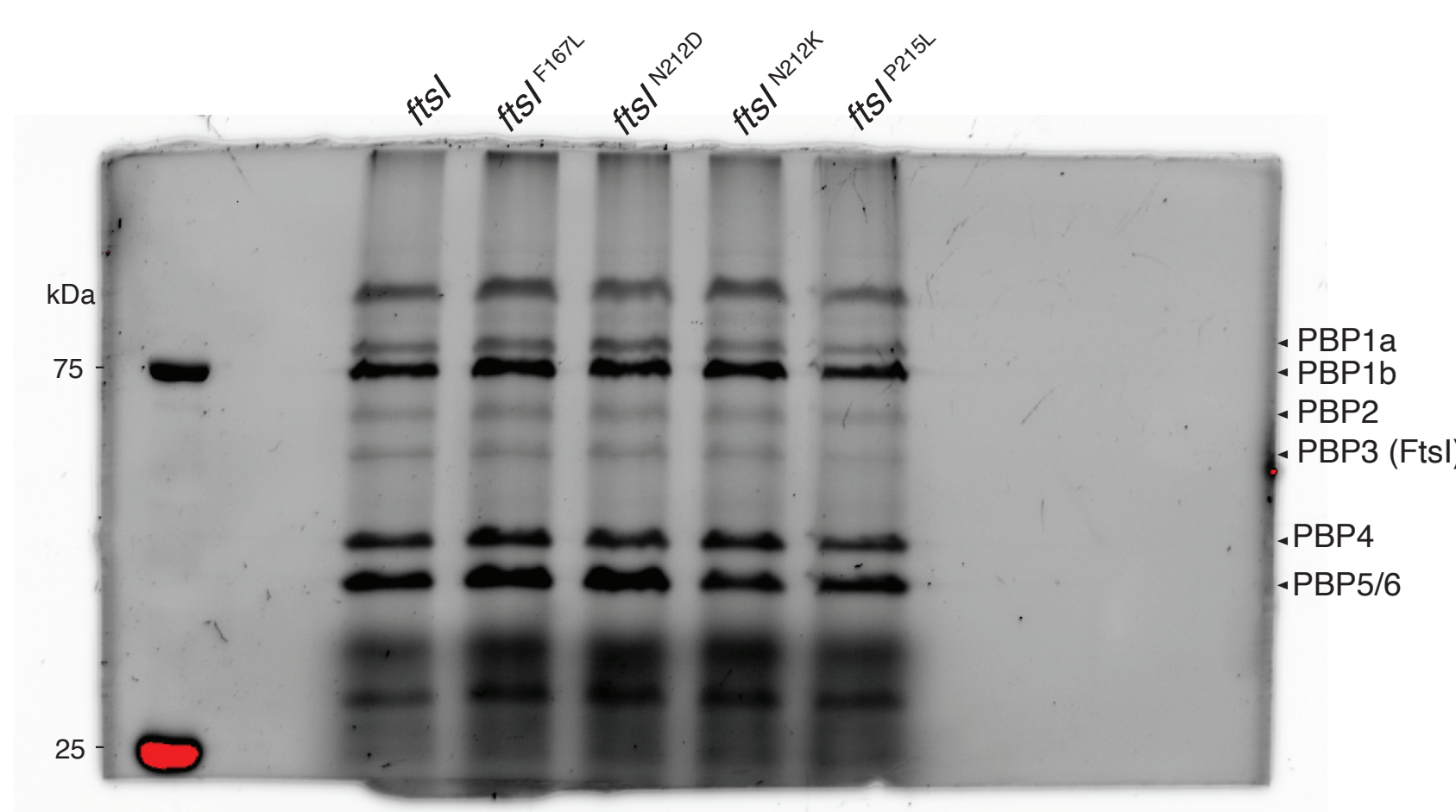

Supplement: Supplementary file 2 [file pgen.1012319.s002.pdf]
